# Exploring the origins of asthma: Lessons from twin studies

**DOI:** 10.3402/ecrj.v1.25535

**Published:** 2014-09-01

**Authors:** Simon Francis Thomsen

**Affiliations:** Department of Respiratory Medicine, Bispebjerg Hospital, Copenhagen, Denmark

## Abstract

This thesis explores the contribution of twin studies, particularly those studies originating from the Danish Twin Registry, to the understanding of the aetiology of asthma. First, it is explored how twin studies have established the contribution of genetic and environmental factors to the variation in the susceptibility to asthma, and to the variation in several aspects of the clinical expression of the disease such as its age at onset, its symptomatology, its *intermediate phenotypes*, and its relationship with other atopic diseases. Next, it is explored how twin studies have corroborated theories explaining asthma's recent increase in prevalence, and last, how these fit with the explanations of the epidemiological trends in other common chronic diseases of modernity.

Asthma is a chronic inflammatory disease of the airways that affects more than 300 million people globally [Global Initiative for Asthma (GINA), 2012; Asher et al., 2006]. Moreover, asthma remains undiagnosed in a significant proportion of the population, making the disease a significant health and economic burden [Nolte et al., 2006]. Clinical manifestations of asthma include wheezing, shortness of breath, chest tightness, and cough. Characteristic triggers of asthma symptoms are exposure to aeroallergens, tobacco smoke, physical exercise, and airway infections. Pathophysiological mechanisms involve airway hyperresponsiveness, reversible airflow obstruction and airway inflammation dominated by eosinophils and mast cells. There is an increase in the number of CD4+ T cells, predominantly T helper 2 (T_H_2) cells, in the airways of asthmatic patients, whereas in normal airways T_H_1 cells predominate. By secreting the cytokines IL-4 and IL-13, which drive IgE production by B cells, IL-5, which is responsible for eosinophil differentiation in the bone marrow, and IL-9, which attracts and drives the differentiation of mast cells, T_H_2 cells have a central role in the asthmatic inflammation. Asthma patients supposedly have a defect in regulatory T cells, which may favour further T_H_2 polarization [Barnes, 2008].

Asthma is a multifactorial disease with complex genetic inheritance. More than a hundred genetic variants positioned throughout the genome have been implicated in asthma susceptibility [Vercelli, 2008]. However, only a subset of these has been replicated in more than a few studies; moreover, the exact mechanisms of interaction between these genotypes and the environment are understood on only a superficial level.

The incidence of asthma is the highest in childhood with a gradual decrease after adolescence. Boys have a greater risk of asthma in early childhood, whereas girls are more frequently affected after puberty. Concomitant sensitization to aeroallergens (*atopy*) is present in the main part of childhood-onset asthma, whereas adult-onset asthma is less related to atopic sensitization [Reed, 2006]. Asthma is closely associated with atopic dermatitis and hay fever, and these three diseases constitute the atopic triad [Bieber et al., 2012].

There is no single diagnostic test that can determine definitely whether a person has asthma. Consequently, the diagnosis is made on the basis of a history of characteristic recurrent airway symptoms concomitantly with an objective verification of airflow limitation such as airway hyperresponsiveness or reversible airflow obstruction [Global Initiative for Asthma (GINA), 2012]. In epidemiological studies, however, where objective tests are not always available, the diagnosis often relies on questionnaire responses [Peat et al., 2001]. Although this method has been shown to have a high specificity, it has only a moderate sensitivity for a clinical diagnosis of asthma [Torén et al., 1993]. It is recognised that asthma is not a single disease but probably constitutes several subtypes of disease, which can be distinguished based on epidemiological, clinical and paraclinical characteristics, and which probably have different causes [Agache et al., 2012].

The prevalence of asthma and other atopic diseases has increased markedly during the past decades and the reasons for this are not fully understood. Asthma is still increasing in many parts of the world, notably in developing countries and this emphasizes the importance of continuing research aimed at studying the aetiological factors of the disease and the causes of its increase in prevalence [Anandan et al., 2010].

Twin studies enable investigations into the genetic and environmental causes of individual variation in multifactorial diseases such as asthma. Thorough insight into these causes is important as this will ultimately guide the development of preventive strategies and targeted therapies. This thesis explores the contribution of twin studies, particularly those studies originating from the Danish Twin Registry, to the understanding of the aetiology of asthma. First, it is explored how twin studies have established the contribution of genetic and environmental factors to the variation in the susceptibility to asthma, and to the variation in several aspects of the clinical expression of the disease such as its age at onset, its symptomatology, its *intermediate phenotypes*, and its relationship with other atopic diseases. Next, it is explored how twin studies have corroborated theories explaining asthma's recent increase in prevalence, and last, how these fit with the explanations of the epidemiological trends in other common chronic diseases of modernity.

## The twin method

The classical twin method examines to what extent genetic and environmental factors contribute to variation in a trait [Martin et al., 1997]. The premise of the twin method is that monozygotic (MZ) twins not only share all their genes, but also their upbringing and early environment. Conversely, apart from their upbringing and early environment, dizygotic (DZ) twins share an average of only 50% of their segregating genes. Therefore, all phenotypic dissimilarity between MZ twins is assumed to be due to non-shared environmental differences between the twins, whereas dissimilarity between DZ twins is assumed to be due both to genetic and non-shared environmental differences. Consequently, if MZ twins are more *similar* for a trait than DZ twins, a genetic contribution to the trait can be inferred [Cardno & McGuffin, 2002].

For quantitative traits the similarity between twins is expressed as an intra-class correlation between the phenotypic scores of the twin pairs. This correlation is calculated from the covariance (COV) between the two trait values (e.g. the level of IgE) for each twin pair. For categorical traits (diseases), e.g. asthma, the similarity between twins is measured by the concordance rate. This rate denotes the probability that one twin is affected given the co-twin is affected [McGue, 1992]. The *probandwise* concordance rate is calculated as follows:

Probandwise concordance rate=2C/(2C+D), where

C=number of concordant pairs (both twins affected) and D=number of discordant pairs (one twin affected).

Twin similarity for diseases can also be expressed as a *polychoric correlation* assuming a normally distributed liability to the disease within the population [Falconer & Mackay, 1996]. The polychoric correlation then represents the correlation in the risk of the disease between the twins and its calculation is based on the relative frequencies of the different sectors of the bivariate normally distributed liabilities for the twin pairs, i.e. the number of concordant, discordant, and unaffected pairs.

The observed similarity between twins for a trait can be used to partition the variance of that trait into genetic and environmental components. According to quantitative genetic theory, the phenotypic variance (P) of a trait can be decomposed into genetic (G) and environmental effects (E) [Neale & Cardon, 1992; Evans et al., 2002]:

1 P=G+E

Or expressed in terms of variance (σ^2^):

2 $$\sigma_{\text{P}}^{\text{2}}=\sigma_{\text{G}}^{\text{2}}+\sigma_{\text{E}}^{\text{2}}$$

The effect of genes can be further decomposed into variance arising from loci contributing linearly (additive genetic effects, *A*) and non-linearly (non-additive genetic effects, *D*) to the trait variance. The A component represents the effect of alleles that independently of other alleles and in an additive manner influence the phenotypic variance, whereas the D component represents the effect of interacting alleles, either from the same locus (genetic dominance) or from separate loci (epistasis). The effect of the environment can be decomposed into variance arising from influences common to members of the same family (influences that increase the similarity between household members, shared environment, *C*), and variance arising from influences unique to individuals (influences that result in differences between family members, non-shared environment, *E*). The E component also includes variance due to measurement error. This gives:

3 $$\sigma_{\text{P}}^{\text{2}}=\sigma_{\text{A}}^{\text{2}}+\sigma_{\text{D}}^{\text{2}}+\sigma_{\text{C}}^{\text{2}}+\sigma_{\text{E}}^{\text{2}}$$

Based on the assumptions of the twin method, the expectations for the covariance between MZ and DZ twins can be derived as follows:

4 $${\text{COV}}_{\text{MZ}}=\sigma_{\text{A}}^{\text{2}}+\sigma_{\text{D}}^{\text{2}}+\sigma_{\text{C}}^{\text{2}}$$

5 $${\text{COV}}_{\text{DZ}}=0.5\sigma_{\text{A}}^{\text{2}}+0.25\sigma_{\text{D}}^{2}+\sigma_{\text{C}}^{2}$$

The proportion of the variance of a trait that arises due to additive genetic differences (the A component) is termed the narrow-sense heritability (or simply the heritability, *h*^*2*^), whereas the proportion of variance of a trait that arises due to both additive and non-additive genetic differences (the A+D components) is termed the broad-sense heritability (*H*^*2*^). Therefore, by definition, the heritability is a ratio of variances and can change according to changes in either the nominator (total genetic variance) or the denominator (total phenotypic variance) [Visscher et al., 2008].

**Fig. 1:**
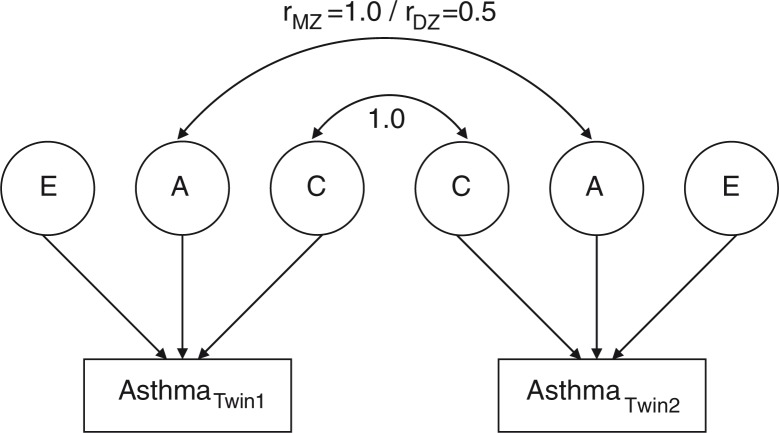
Classical twin model of asthma. Note: A classical twin model showing latent additive genetic effects (A), shared environmental effects (C), and non-shared environmental effects (E) on asthma measured in a pair of twins. The arrows pointing from each of the latent factors to the trait (asthma) account for the variation observed in asthma. Additive genetic effects are correlated 1.0 across MZ twins and 0.5 across DZ twins, whereas shared environmental effects, by definition, are correlated 1.0 both across MZ and DZ twins. Non-shared environmental effects are uncorrelated across twin pairs. In cases where non-additive genetic effects can be assumed to contribute to the trait variance (MZ correlation above twice the DZ correlation) the shared environmental variance component is substituted with a non-additive genetic variance component. Non-additive genetic effects are correlated 1.0 across MZ twins and 0.25 across DZ twins.

For most human traits it is reasonable to assume that all four sources of variance (A, D, C, and E) act simultaneously. However, components C and D are not identified with the same model in studies that include only twins reared together [Keller & Coventry, 2005]. Therefore, the likelihood of the observed data in a twin study is typically determined with a saturated model that includes only components A, C, and E whenever there is evidence that shared environment could influence the trait variance (MZ correlation below twice the DZ correlation), or A, D, and E whenever there is evidence that genetic non-additivity could influence the trait variance (MZ correlation above twice the DZ correlation). These relationships can be depicted in path diagrams showing loadings of genetic and environmental factors on the trait (Figure 1). The relative contribution of the individual parameters to the trait variance is determined by solving equations (3), (4), and (5) preferably by using maximum likelihood estimation [Neale et al., 2006]. The significance of the contribution of each of the variance components can be estimated by comparing the fit of the full ACE or ADE model with nested models (AE, DE, CE and E models).

The twin method can be extended to include several traits simultaneously in order to estimate the extent to which the same genetic and environmental factors influence different traits. This analysis uses the information that lies in the measured covariance between different traits. Notably, a larger covariance (resemblance) between two traits across MZ twins compared with DZ twins indicates that those two traits share genetic variance, i.e. that the same genetic factors can be assumed to influence both traits [Posthuma et al., 2003]. The underlying relationship between pairs of traits can be expressed as the genetic and environmental correlations, respectively, between the traits. These statistics are calculated as the genetic or the environmental covariance between two traits divided by the square root of the product of the genetic or the environmental variances of those two traits, respectively.

Several other extensions to the classical twin method exist. For example, an extension to the bivariate twin model is the *direction of causation model*, which can be used to resolve the direction of causation between two traits given cross-sectional twin data, particularly if the measured traits have different variance structures [Duffy & Martin, 1994; Gillespie & Martin, 2005]. This model can be used to test whether trait A is more likely to “cause” trait B, or whether trait B is more likely to “cause” trait A.

Another twin study design is the *co-twin control study*, which is a matched case-control study design that uses twin pairs discordant for an exposure or an outcome. Particularly because twin pairs are inherently matched on genetics and several other factors relating to early life and upbringing, this type of study design can be used to study the relationship between exposure and outcome and whether this is influenced by genetic or non-genetic confounding factors [Duffy, 2000].

Twin studies are based on a number of assumptions, the most important being: (i) MZ and DZ twins share environmental exposures in the rearing (and intrauterine) environment to the same extent - *the equal environments assumption*; (ii) MZ twins are genetically identical; (iii) the prevalence of the trait under study is the same in MZ twins, DZ twins, and singletons; and (iv) random mating (*panmixia*). Violations of these assumptions may compromise the validity and generalisability of twin studies [Cardno & McGuffin, 2002; Rose, 2006].

### The Danish Twin Registry

The most systematic and least biased way to recruit twins for research is through national registries. The Danish Twin Registry is the oldest nationwide twin registry in the world [Skytthe et al., 2011]. It was established in 1953 with the aim of studying genetic and environmental influences on a variety of common chronic diseases. It contains data on Danish twins born after 1870 and at present includes over 85,000 pairs. All twins have been ascertained independently of the traits studied. Separate rounds of ascertainment have been made to form the entire registry, which can be divided into cohorts born 1870–1930, 1931–1952, 1953–1982, 1983–2000, and 2001–present.

The populations mainly used to study asthma in the Danish Twin Registry, and which form the population described in this thesis, comprise twins born 1931–2000. The oldest part of this cohort corresponded to 70% of all twin births in Denmark; whereas after 1968, when the Civil Registration System was introduced in Denmark, there is complete ascertainment of twin pairs with both members live born. For a detailed description of the recruitment of these cohorts, the reader is referred to previous publications [Hauge et al., 1968; Kyvik et al., 1995; Skytthe et al., 2002]. Zygosity of the twins is determined using four questions of similarity and mistaken identity between the twins, which, in adults, assign zygosity correctly in more than 95% of the cases compared with genetic marker information [Christiansen et al., 2003].

In 1994 (individuals born 1953–1982), in 2002 (individuals born 1931–1952 and 1953–1982), and in 2003 (individuals born 1983–2000), the twins participated in multidisciplinary questionnaire-based studies concerning health and lifestyle wherein a history of asthma [Ferris, 1978] and other health attributes and socio-demographic characteristics were recorded. Furthermore, in 2004, a sample of twins from the cohort born 1953–1982, who were living in the eastern part of Denmark, and in whom at least one member of each twin pair had reported a history of asthma in the questionnaire study in 2002, underwent a clinical examination. This examination included an interview on asthma [Global Initiative for Asthma (GINA), 2012] and rhinitis [Bousquet et al., 2001], as well as paraclinical tests supportive for asthma and allergy such as spirometry [Nysom et al., 1997], methacholine challenge [Yan et al., 1983], measurement of exhaled nitric oxide (FeNO) [Kharitonov et al., 1997], serum total IgE, and skin prick test (SPT) to common aeroallergens [Dreborg, 1987]. It was estimated that among those who reported a history of asthma in the questionnaire-based study, 11% were subsequently diagnosed not to have (or to have had) asthma based on the clinical interview and the findings of the clinical examination. Conversely, of those who reported no history of asthma in the screening questionnaire, a total of 12% were diagnosed with asthma at the clinical examination, giving a misclassification rate of asthma of approximately 12% (sensitivity of the asthma screening question, 88%; specificity, 89%). Table 1 provides an overview of the cohorts studied and presented in this thesis.

**Table 1:** Danish twin studies of asthma

Population (birth year)	Year studied	Age (years)	Participants (%)	Males (%)	Females (%)	Intact pairs	References
1931–1952							
Questionnaire study	2002	50–71	13,649 (75)	6,415 (47)	7,234 (53)	4,240	[I, II, VI, IX]
1953–1982							
Questionnaire study	1994	12–41	29,183 (86)	14,074 (48)	15,106 (52)	11,231	[IV, V]
Questionnaire study	2002	20–49	21,133 (75)	9,431 (47)	11,702 (53)	7,201	[I, II, III, VI, IX]
Clinical study	2004	21–51	575 (67)	240 (42)	335 (58)	256	[III]
1983–2000							
Questionnaire study	2003	3–20	19,748 (68)	9,896 (50)	9,850 (50)	9,694	[I, V, VI, VII, VIII]

Three individuals from 1994 and two individuals from 2003 were of unknown sex. Intact pairs are twin pairs with complete data on asthma.

## Genetic epidemiology of asthma

It has long been known that asthma aggregates within families. As early as in 1650 Sennertus observed that asthma was present in successive generations of his wife's family [Wiener et al., 1936]. In 1868 Salter reported that in a sample of 217 asthma patients, 39% had a positive family history of the disease [Salter, 1868], and in 1920 Adkinson found that among 400 asthma patients from Boston, 48% had a familial predisposition [Adkinson, 1920]. In 1916 Cooke & van der Veer, and in 1924 Spain & Cooke noted in another population from the United States that a family history of asthma was present in 58% of patients with the disease compared with only 7% in a control population [Cooke & van der Veer, 1916; Spain & Cooke, 1924]. Moreover, asthma was found to occur at an earlier age in those who had two affected parents compared with those who had only one affected parent, and even earlier than in subjects with sporadic asthma [Spain & Cooke, 1924]. In 1952 Schwartz obtained extensive pedigree data based on 191 asthma probands from Copenhagen and observed that asthma occurred with increased frequency in family members according to their degree of relatedness with the index patient [Schwartz, 1952].

### Genetic and environmental influence on asthma

Twin studies have been pivotal for establishing empirical prognostic values for the recurrence risk of asthma within families. Already in 1936 Spaich & Ostertag studied 2,500 German twin pairs and found probandwise concordance rates of asthma of 0.44 in MZ twins and 0.13 in DZ twins [Spaich & Ostertag, 1936]. In 1956 Harvald & Hauge performed a study of 1,900 twin pairs from the eastern part of Denmark and found a probandwise concordance rate of hospital diagnosed asthma of 0.50 in MZ twins and 0.17 in DZ twins [Harvald & Hauge, 1956]. In 1970 Edfors-Lubs studied 6,996 Swedish twin pairs and found concordance rates for asthma of 0.19 in MZ twins and 0.05 in DZ twins [Edfors-Lubs, 1971]. The two latter studies were among the first to represent an era during the second half of the last century when twins were systematically recorded in national registries, primarily in Scandinavia. As a result, estimates of recurrence risks of asthma were more reliable because the ascertainment of the individual twins was independent of the disease status of the co-twins.

**Fig. 2:**
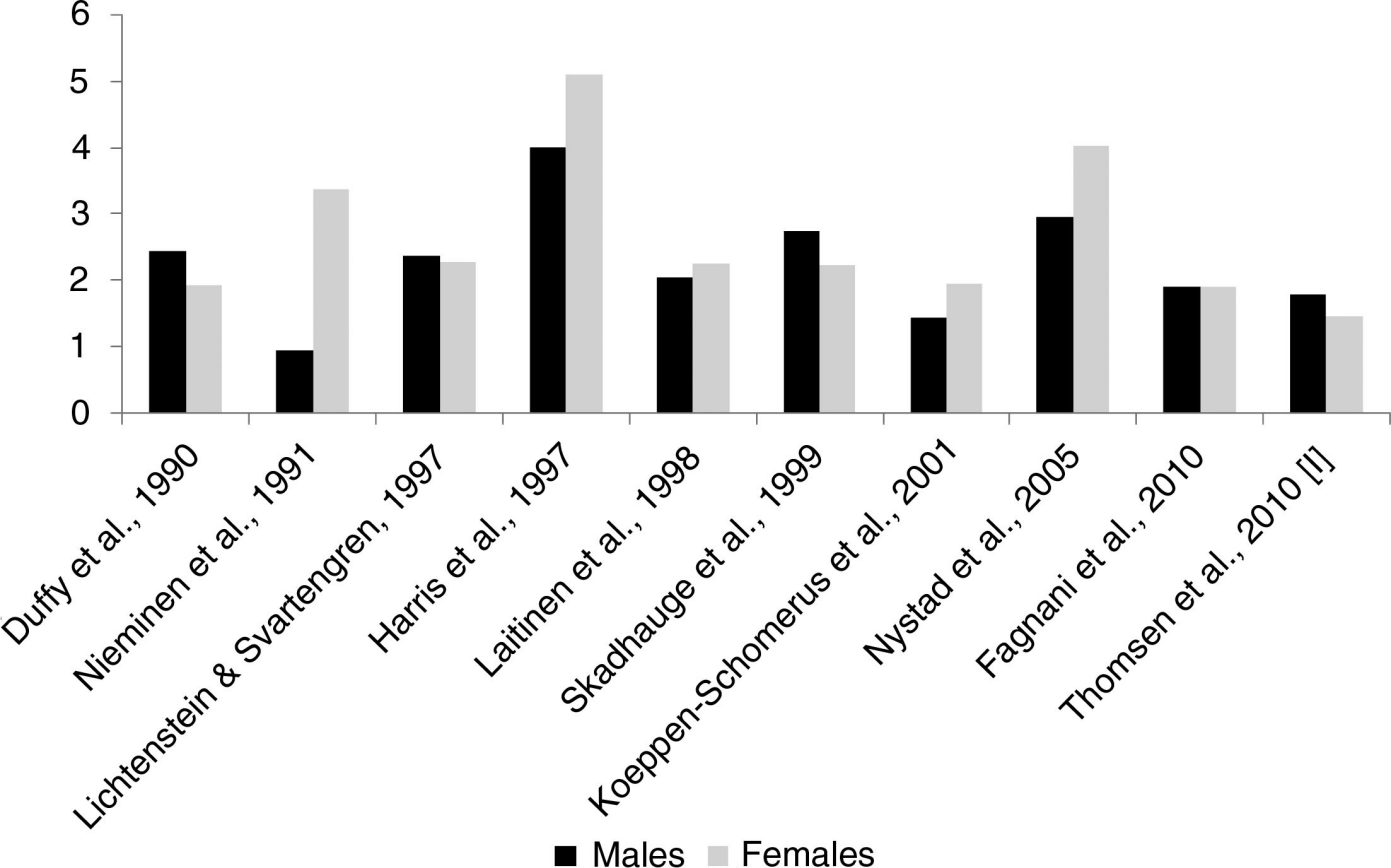
Ratio between MZ and DZ concordance rates for asthma obtained from recent population-based twin studies.

Several registry-based twin studies of asthma have been performed (Figure 2). The largest twin study of asthma performed to date is a questionnaire-based study of 21,135 Danish twin pairs, 3–71 years of age [I]. Due to its size it provided the opportunity to study the variation in the influence of genetic and environmental factors on asthma over the lifespan. In that study the overall probandwise concordance rate of self-reported asthma was 0.53 in MZ twins and 0.28 in DZ twins, consistent with a ratio between concordances in MZ and DZ twins of 1.89 [I]. However, the ratio between concordance rates in MZ and DZ twins differed between age groups and sexes and was the highest among 3–20-year-old individuals (1.88 in males and 1.74 in females) and among 20–49-year-old individuals (2.65 in males and 1.48 in females) compared with 50–71-year-old individuals (1.08 in males and 1.50 in females), indicating an influence of age- and sex-specific genetic effects on asthma [I]. Specifically, the influence of genetic factors was shown to be the most pronounced in the youngest age group but to decrease over the lifespan, particularly among males. Other population-based twin studies have shown that the ratio between MZ and DZ concordance rates is consistently higher than 1.0 but with some variation between countries. While most studies find a ratio between MZ and DZ twins of around 2.0, consistent with an additive genetic model of inheritance, some studies, particularly from Norway [Harris et al., 1997; Nystad et al., 2005], show very high ratios between MZ and DZ concordance rates (up to 5.0), suggesting genetic dominance or epistasis, whereas other studies show ratios below 2.0, indicating a role of shared environment [I; Nieminen et al., 1991; Koeppen-Schomerus et al., 2001; Fagnani et al., 2008]. One small twin study combining data from the United States and Finland found similar concordance rates for asthma in 53 MZ twins *reared apart* (concordance rate=0.89) compared with 110 MZ twins *reared together* (concordance rate=0.80), suggesting that shared environment has very little effect on the development of asthma [Hanson et al., 1991].

**Fig. 3:**
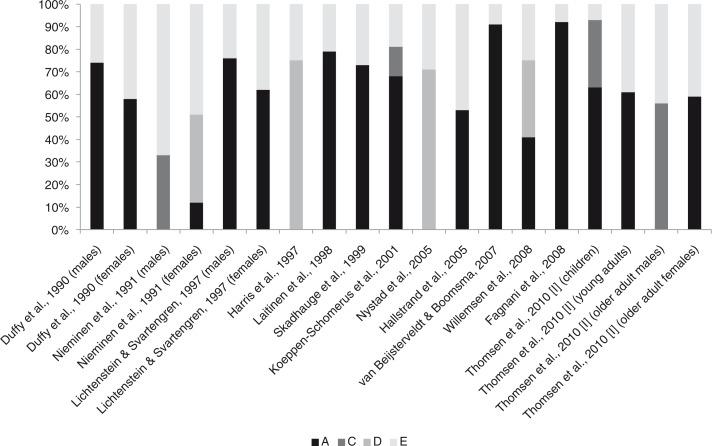
Variance decomposition of asthma obtained from recent population-based twin studies. Note: Proportion of variance (%) in asthma susceptibility due to additive genetic effects (A), shared environmental effects (C), non-additive genetic effects (D), and non-shared environmental effects (E). Variance components sum to 100%. Unadjusted variance components were recalculated from the raw data for Nieminen et al., 1991, using the statistical software Mx [Neale et al., 2006].

Despite heterogeneity between different twin studies regarding diagnostic criteria of asthma, age and year of examination, and country of origin, the collective evidence is consistent with asthma being a highly heritable disease with genetic factors accounting for approximately 60–80% of its susceptibility and with only a modest or no effect attributable to environmental effects shared between family members (Figure 3). However, even though heritability estimates of asthma are high, we should bear the possibility in mind that the heritability could also be overestimated. For example, estimates of asthma heritability in 5-year-old children from the Netherlands [van Beijsterveldt & Boomsma, 2007] and 8–17-year-old children from Italy [Fagnani et al., 2008] are very high (91% and 92%, respectively), as are estimates among children and adolescents from Sweden (76% in boys) [Lichtenstein & Svartengren, 1997] and Finland (87%) [Laitinen et al., 1998]. However, the Finnish estimate of heritability was based on children with parental predisposition to asthma; in children with sporadic asthma the heritability was shown to be much lower [Laitinen et al., 1998]. In fact the Finnish study showed that a model with the same genetic and environmental effects could not account for the observed familial resemblance of asthma both in families with and without asthma, as environmental effects (shared and non-shared) were sufficient to account for the variation in the susceptibility to sporadic asthma [Laitinen et al., 1998]. In addition, the Italian and Swedish studies included only 392 and 1,480 twin pairs, respectively, and these relatively small numbers of twins may have inflated the contribution of genetic effects in these studies. In fact, the inherently low power of the classical twin method to detect effects of shared environment may partly explain the absence of shared environmental influences on asthma found in several former investigations [Neale et al., 1994; Visscher, 2004]. Specifically, the ratio between concordance rates in MZ and DZ twins in the Italian study was 1.91, indicating shared environment, but the study was insufficiently powered to detect this effect, thereby spuriously attributing all familial variation to genetic variation [Fagnani et al., 2008].

A high heritability may also be due to other factors, for example, violations of the equal environments assumption for MZ and DZ twins. This would be the case if MZ twins were sharing their early (intrauterine) environment and upbringing to a greater extent than DZ twins, in which case the MZ concordance would increase relative to the DZ concordance but due to environmental reasons. It is uncertain whether this potential bias of twin studies plays a role in asthma, but in other traits, for example, most common psychiatric disorders, the degree of environmental sharing has not been shown to predict concordance rates [Cardno & McGuffin, 2002]. However, the implications of differences in the intrauterine environment and early life circumstances of MZ and DZ twins for the development of asthma is currently unknown, and such differences could potentially affect the ratio of similarity between MZ and DZ twins [Martin et al., 1997].

Another possible explanation for the high heritability of asthma is gene-environment interaction. In the classical twin model, the proportion of phenotypic variance explained by gene-environment interaction is, unless specifically modelled, automatically included in the genetic variance component, and this would tend to inflate the estimate of genetic variance [Purcell, 2002]. In fact the absence of shared environmental influences on asthma observed in twin studies contrasts with the findings from singleton populations wherein several environmental exposures relating to early life, such as exposure to air pollution [Islam et al., 2009; Schroer et al., 2009], tobacco smoke during pregnancy [Ramadas et al., 2007; Wang et al., 2008], and pollen in the perinatal period [Kihlström et al., 2002], have been shown to be important for asthma development, particularly in individuals with certain genetic polymorphisms.

An exception to the widespread absence of effects of shared environment in twin studies has been observed among Danish twins, in whom 30% of the variation in the susceptibility to asthma among 3–20-year-old individuals was explained by shared environmental factors [I]. Furthermore, among Danish twins, 50–71 years of age, there was no statistically significant contribution of genetic factors to asthma [I]. Instead, shared environment explained 56% of the variation in the susceptibility to asthma in this age group [I]. Additionally, a significant influence of shared environment has been observed among 4-year-old children from the UK [Koeppen-Schomerus et al., 2001]; furthermore, among Finnish men up to 80 years of age, there was no evidence of a contribution of genetic factors to asthma [Nieminen et al., 1991]. However, the effect of shared environment observed at the extreme ends of the age spectrum in these studies may reflect other factors. For example, in the study from the UK, the diagnosis of asthma was based on medication use and therefore could reflect diagnostic mix-up with wheezy bronchitis, which has an infectious origin and therefore would tend to affect DZ co-twins to the same extent as MZ co-twins, thereby diluting the difference between zygosity groups. In the Finnish twin study, the asthma diagnosis relied partly on reimbursement of medication for asthma, which may pose similar problems. Furthermore, asthma in older adults may reflect smoking-related respiratory symptoms, such as chronic bronchitis or chronic obstructive pulmonary disease (COPD), which have been shown to have a lower heritability than asthma. For example, a large twin study from Sweden found a heritability of chronic bronchitis of only 40% [Hallberg et al., 2008], whereas the heritability of COPD has been estimated to be 63% in Danish and 61% in Swedish adult twins [Ingebrigtsen et al., 2010].

Taken together, the heritability of asthma has been shown to be substantial. This is in accordance with the overwhelming evidence of many genes regulating the pathogenesis of the disease [Vercelli, 2008]. Nevertheless, methodological factors relating to study design and properties of the classical twin method may have resulted in overestimation of heritability. Furthermore, a high heritability does not preclude an important contribution of environmental factors to asthma susceptibility. Accordingly, the interpretation of heritability estimates must be rooted in the context of a permissive environment. Most twin studies have involved adolescents and adults of European ancestry, and estimates of asthma heritability in very young children and in different ethnic groups are lacking in the literature. Future twin studies should focus on these issues and also include more extensive clinical data as most of our knowledge of asthma heritability stems from self-reported questionnaire-based measures of disease.

**Fig. 4:**
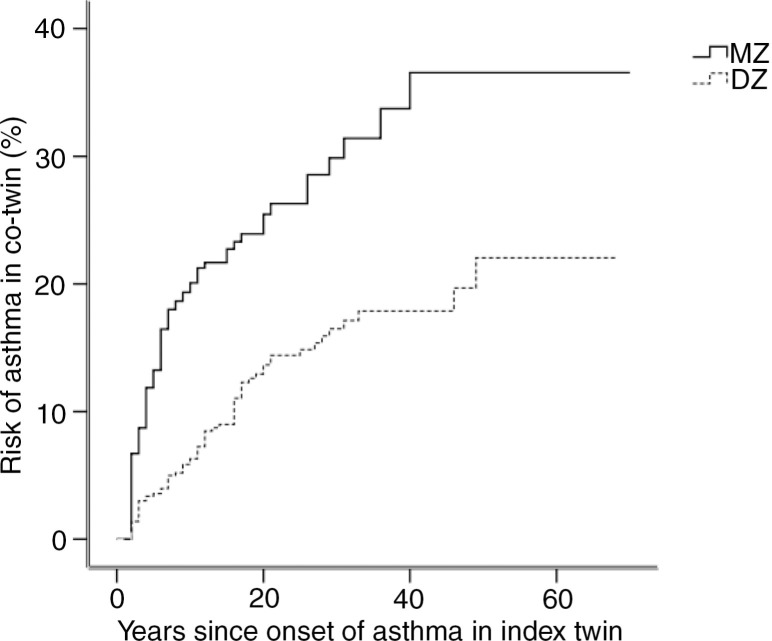
Risk of asthma in the co-twin as a function of the age at onset of asthma in the index twin in Danish twins, 20–71 years of age. Modified from [II].

While most twin studies have concerned the variation in susceptibility to asthma, only a few studies have examined more elaborated aspects of the phenotypic expression of the disease. For example, the age at onset of asthma has been examined in only one previous twin study [II]: among Danish twins the age at onset of self-reported asthma in a twin was significantly influenced by the age at onset of asthma in the co-twin [II]. Notably, the correlation between the ages at onset of asthma was higher in MZ twins than in DZ twins (0.37 vs. 0.09), predominantly among males, where it was five times higher (0.42 vs. 0.08) compared with females where it was only two times higher (0.34 vs. 0.18). These findings indicate that the degree of genetic relatedness between family members dictates the expected waiting time to onset of asthma in the second family member after onset of asthma in the first (Figure 4). Moreover, the stronger a person's genetic predisposition for asthma, the earlier he or she develops the disease, i.e. if the first member of a twin pair develops asthma at an early age, then the co-twin also has a higher risk of developing asthma at an early age, whereas late-onset asthma is less dependent on asthma status of the co-twin. Among Danish twins, this effect was more prominent in MZ twins than in DZ twins [II], corroborating Spain & Cooke's early finding that asthma develops earlier in those with homozygous parents compared with those who have heterozygous parents [Spain & Cooke, 1924]. A total of 34% of the variation in the age at onset of asthma in Danish twins was explained by genetic factors (the heritability of age at onset of asthma), whereas the remaining 66% of the variation was explained by environmental factors [II].

Genetic variation underlying the age at onset of asthma has been traced to diverse chromosomal loci. Specifically, Bouzigon and colleagues found evidence of two regions (5q13 and 1p31) with suggestive linkage to time to onset of asthma in French families [Bouzigon et al., 2007]. Furthermore, a region on 7q showed suggestive linkage to asthma in the same population but with different risks according to the age at onset of disease [Dizier et al., 2001]. Hizawa et al. found that the *-28G* allele of the *RANTES* promoter region at chromosome 17q increased the risk of late-onset asthma (>40 years of age) compared with early- and middle-age-onset asthma in Japanese individuals [Hizawa et al., 2002]. In contrast, a genome wide association (GWA) study revealed that the 17q12-21 region (*IKZF3-ZPBP2-GSDMB-ORMDL3* region) is predominantly a childhood-onset asthma locus, with modified effects through environmental tobacco smoke exposure [Dijk et al., 2013]. Despite these findings, it is not clear whether the genetic heterogeneity underlying asthma at different ages also causes the clinical heterogeneity that characterizes asthma with different ages at onset. For example, it is not fully understood whether sex differences in asthma risk at different ages can be explained by genetic factors. Additionally, understanding is lacking about whether the difference in long-term prognosis in relation to decline in lung function or the degree of airflow obstruction that characterizes asthma with onset at different ages can be attributed to genetics. Furthermore, the different association with atopy and type of airway inflammation (eosinophilic or neutrophilic) that characterizes asthma with different ages at onset may also be explained by genetic factors and deserves further exploration. Hitherto, most twin studies of asthma have been cross-sectional or retrospective and therefore future twin studies should address the variation in the timing of asthma-onset using clinical prospective cohorts with repeated measurements of asthma.

Only a few twin studies have concerned specific asthma symptoms, and only one twin study has estimated the genetic and environmental influences on asthma symptomatology using clinically validated interview-based data [III]. In this study of Danish twin pairs, 21–51 years of age, the overall symptomatic severity of asthma diagnosed by the Global Initiative for Asthma (GINA) classification system [Global Initiative for Asthma (GINA), 2012] was correlated 0.37 among 38 MZ twin pairs and 0.11 among 21 DZ twin pairs concordant for asthma [III]. Individual asthma symptoms, particularly wheezing (0.39 in MZ twins vs. 0.07 in DZ twins), shortness of breath (0.37 vs. −0.20) and cough (0.30 vs. 0.01) were also correlated to a higher extent among MZ than among DZ twin pairs concordant for asthma, whereas chest tightness was not (−0.01 vs. 0.04). It was found that genetic factors explained 24% of the variation in overall asthma symptom severity, whereas non-shared environment accounted for the remaining 76%, indicating that random variation and variation due to specific environmental exposures account for the main part of the variation in asthma symptom severity between individuals. Statistically significant genetic components were also observed for variation in wheezing and shortness of breath with heritabilities of 12% and 17%, respectively, but not for variation in chest tightness (0%) and cough (1%) [III]. However, the fact that the twins were ascertained through a proband with asthma may have resulted in a lower heritability of asthma symptoms, although statistical measures were taken into account for this effect. The Danish twin study was also the first to estimate the *correlation* between genetic and environmental effects on different asthma symptoms [III]. Specifically, among Danish twins, genetic effects on wheezing and shortness of breath were found to correlate to unity, and environmental effects for these two symptoms correlated substantially (Table 2). In contrast, genetic and environmental correlations between the other asthma symptoms (e.g. chest tightness and cough) were modest, and in general, the different specific asthma symptoms seemed to arise from distinct genetic determinants [III].

**Table 2:** Genetic and environmental correlations between asthma symptoms and intermediate asthma phenotypes in a sample of 575 Danish adult twins

	Over	Whee	Short	Chest	Cough	Rhin	FEV_1_	FVC	FE/FV	DRS	FeNO	IgE	SPT	HDM
Overall symptom severity		0.50***	0.47***	0.26***	0.86***	0.20*	−0.22*	−0.20*	−0.18*	0.32***	0.09	0.08	0.22*	0.15
Wheezing	0.68**		0.64***	0.17	0.24**	0.17*	−0.10	−0.09	−0.07	0.34***	0.16*	0.11	0.18*	0.19*
Shortness of breath	0.72**	1.00***		0.17**	0.20*	0.14	−0.26***	−0.13	−0.19*	0.44**	0.12	−0.05	0.15*	0.11
Chest tightness	0.61	−0.15**	0.27		0.27***	−0.06	−0.02	−0.04	−0.03	0.11*	−0.03	0.00	0.01	−0.01
Cough	1.00***	0.52	0.58*	0.68*		0.12	−0.10	−0.01	−0.23**	0.21*	0.12	0.05	0.22**	0.05
Rhinitis severity	0.02	0.63*	0.37	−0.43	0.00		0.03	−0.04	0.06	0.27**	0.22*	0.12	0.31***	0.12
FEV_1_	−0.27	−0.40	−0.45**	0.07	−0.04	0.02		0.75***	0.53***	0.35**	−0.02	−0.02	0.00	0.01
FVC	**−**0.01	−0.27	−0.44**	−0.59	−0.07	−0.01	0.90***		0.13	−0.01	0.04	−0.06	−0.02	−0.02
FEV_1_/FVC	0.09	0.02	0.14	−0.37	0.22	0.16	−0.30**	0.67***		0.46***	−0.11	0.07	0.01	0.05
DRS_methacholine_	0.25	0.62**	0.43*	−0.34	0.22	0.09	0.39**	−0.37**	0.26*		0.34**	0.24*	0.27*	0.12
FeNO	0.15	0.13	0.09	−0.50	−0.03	0.31	0.29*	0.19	−0.02	0.20		0.08	0.29**	0.26**
Serum total IgE	−0.03	0.32	0.11	0.90	−0.03	0.40*	0.13	−0.03	0.00	0.24*	0.37***		0.35***	0.28**
Positive SPT	−0.12	0.24	0.10	−0.07	−0.23	0.90***	0.21	0.08	0.14	0.26*	0.41***	0.34**		0.46***
HDM sensitization	−0.17	0.21	0.11	−0.63	−0.22	0.54***	0.18	0.04	0.17	0.31*	0.44***	0.25*	0.73***	

Genetic correlations are below the diagonal; environmental correlations are above the diagonal.

* p<0.05

** p<0.01

*** p<0.001 (highlighted in bold print). Modified from [III].

A few other small twin studies have examined the familial aggregation of asthma symptoms [Sarafino & Goldfedder, 1995; Miller et al., 2005]. Specifically, among 2–20-year-old twins from the United States, the frequency and intensity of asthma symptoms correlated 0.63 in 23 MZ twin pairs concordant for asthma, which significantly exceeded the correlation of −0.12 in 13 DZ twin pairs [Sarafino & Goldfedder, 1995]. Moreover, a French family study found a correlation of 0.23 between first-degree relatives for a clinical asthma severity score comprised of asthma attack frequency in the past year, persisting symptoms between attacks, and hospitalisation for asthma within the past year [Pin et al., 2002]. However, the risk of asthma was not increased in relatives according to asthma severity in the index case, indicating that the risk of asthma in a relative was independent of the severity of asthma in the family members.

A genetic liability to the clinical severity of asthma is supported by several molecular genetic studies. A suggestive association was found for a variant on chromosome 5, situated within the *TSLP* gene, among patients with severe asthma [Moffatt et al., 2010]. Moreover, several other studies have associated genetic variants with asthma severity. For example, polymorphisms within the beta2-adrenergic receptor gene [Weir et al., 1998; Holloway et al., 2000], the *IL-4* gene [Rosa-Rosa et al., 1999; Chouchane et al., 1999; Sandford et al., 2000], and the *TGF-β1* and *CD14* genes [de Faria et al., 2008] have been shown to play a role in asthma severity.

Taken together, genetic factors influence the variation in the clinical expression of asthma; moreover, a particular symptomatology in an asthma patient seems to predict the same pattern of disease expression in an affected relative. However, there is also evidence of substantial genetic heterogeneity within the clinical expression of asthma. The varying heritability of, and the varying correlation between, individual asthma symptoms suggest that asthma is not a genetically homogeneous disease, i.e. different symptoms seem to have different genetic backgrounds and which symptoms occur may depend on the genetic make-up of the patients. Potentially, this observation has implications for gene identification [van der Sluis et al., 2013], and studies in which asthma symptoms are separately subjected to genetic analyses (with measured genotypes) would be very interesting.

### Intermediate asthma phenotypes

Intermediate asthma phenotypes *(endophenotypes)* are objectively measurable traits that manifest to a variable degree irrespective of the presence of asthma and which are found in non-affected family members of asthma patients more often than in the general population [Gottesman & Gould, 2003]. There are several intermediate phenotypes associated with asthma; however, mainly lung function and allergic sensitization have been studied in twins. Specifically, among 256 Danish twin pairs aged 21–51 years, the heritability of forced expiratory volume in one second (FEV_1_), and forced vital capacity (FVC) was 68% and 58%, respectively, whereas a lower heritability was found for FEV_1_/FVC ratio (22%) (Table 3) [III]. Furthermore, in a large sample of older adult Danish twins (4,314 pairs) the heritability of several measures of pulmonary function was estimated: FEV_1_ (61%), FVC (55%), and peak expiratory flow (PEF) (43% in males and 0% in females) but with 25% of the variation in female PEF explained by shared environmental factors [Ingebrigtsen et al., 2011]. In Australian twins (>700 pairs) and their families, heritability estimates of lung function were fairly similar compared with the Danish studies, at least for FEV_1_ (71%) but not for FEV_1_/FVC, where it was higher (51%) [Ferreira et al., 2006]. A study of 192 adult Hungarian and American twin pairs found a heritability of FEV_1_ of 73% and of FVC of 68% [Tarnoki et al., 2013a]. In a small adult twin sample from the Netherlands (103 pairs), the heritability of FEV_1_ and FVC was 83% and 72%, respectively, whereas the heritability of FEV_1_/FVC was 61% [Wu et al., 2010]. A meta-analysis of twin- and family studies published up until 1999 found that the heritability of several pulmonary function indices, particularly FEV_1_ and FVC, was highly variable and depended on several factors such as age, sex, body composition, ethnic background, individual and passive smoking history, familial predisposition to, or presence of respiratory disease, as well as methodological issues - specifically adjustment for covariates and sampling strategy [Chen, 1999]. Moreover, a longitudinal study of Finnish female twins, 63–76 years of age showed that the proportion of variance in FEV_1_/FVC explained by environmental effects increased remarkably during a three-year period [Hukkinen et al., 2011]. This finding is consistent with a study of PEF measured at four time points in Swedish twins above 80 years of age, which showed that the genetic variance in PEF was attributable to genetic transmissions from prior time points, whereas the specific environmental variance in PEF at each time point was mainly due to environmental innovations [Vasilopoulos et al., 2010].

**Table 3:** Heritability of intermediate asthma phenotypes in a sample of 575 Danish adult twins

	Heritability (%)
FEV_1_	68*
FVC	58*
FEV_1_/FVC	22
DRS_methacholine_	43*
FeNO	67*
Serum total IgE	81*
Positive SPT	54*
HDM sensitization	6
Serum tryptase	82*

Serum tryptase was studied by Sverrild et al., 2013.

* p<0.001. Modified from [III].

Collectively, the results from the Danish and other twin studies imply that genetic factors contribute to the stability of pulmonary function over time, whereas environmental factors contribute to its change (i.e. reduction). One such factor may be smoking, which has been shown to modify the genetic influence on FEV_1_ in adult twins [Zhai et al., 2007]. Interestingly, two twin studies of children [Yu et al., 2007] and adults [Højland et al., 2004], respectively, have found that the expected spirometric values in twins and singletons are comparable, indicating that the findings from twin studies regarding lung function are applicable to the population as a whole.

Another important question addressed in twin studies is whether the inherited tendency to become allergic (to be atopic) extends to specific allergens. Several twin studies of recent and older dates have shown that the risk of atopy as well as the variation in serum total IgE is under genetic control, with both traits having a moderate to high heritability [Bazaral et al., 1974; Blumenthal & Bonini, 1990]. Interestingly, a study of 57 Danish twins showed that, after exclusion of materno-fetal transfer of IgE, as much as 80% of the variation in cord blood IgE was ascribable to genetic effects, indicating a substantial influence of genetic factors for this trait already before the onset of clinical allergic disease [Husby et al., 1996]. However, follow-up of this cohort showed that the correlation between cord blood IgE and serum total IgE at age 6–9 years was close to zero, indicating that different effector mechanisms may be operating at different ages [Jacobsen et al., 2001]. Wüthrich and colleagues studied 50 twin pairs from Switzerland with at least one atopic proband and found that the concordance for atopy was 0.57 in MZ twins compared with only 0.20 in DZ twins, consistent with a clear role of heredity for the tendency to become sensitized [Wüthrich et al., 1981]. However, the specific reagin production, as measured by a radioallergosorbent test (RAST) or skin test, was similar in MZ and DZ twins, indicating that although the tendency to IgE production is genetically determined, its specificity is governed mainly by environmental influences [Wüthrich et al., 1981]. In other words, the tendency to become allergic is inherited, whereas a person's specific allergies depend on which allergens he or she encounters. For example, in a study of 58 twins, aged 0–11 years from the United States, there was significant excess in the similarity between MZ compared with DZ twins for overall atopy and total IgE, but in the subgroup of twins where both had allergy, both twins had skin test reactivity to the same allergen in only two out of 15 DZ sets, and in none of 9 MZ sets [Yilmaz-Demirdag et al., 2010]. Furthermore, analysis of the specific immune response, as measured by RAST and SPT, to ragweed, grass, and mould in 163 MZ and 132 DZ twin pairs from the United States and Finland reared together and apart showed no significant differences either when comparing MZ twins reared apart with DZ reared apart, or when comparing MZ twins reared together with DZ twins reared together [Hanson et al., 1991]. These results indicate that sensitivity to particular allergens may be influenced more by environmental factors than by genetic factors [Duffy et al., 1998]. In 282 adult female MZ twin pairs from the UK tested for specific IgE to house dust mite (HDM), grass, and cat, there were substantial differences in the individual patterns of allergen sensitization within the twins, indicating a role of random environmental events in the determination of specific allergen reactivity [Strachan et al., 2001]. Of further note, in 74 MZ and 68 DZ twin pairs from Australia concordant for HDM allergy, there were significant differences in the concordance between MZ and DZ twins for only two IgE responses out of 36 specific IgE-binding HDM components tested. In MZ twins, concordance never exceeded 0.67 for any epitope, and most MZ twins recognized epitopes their co-twin did not, indicating that genetic control of overall atopy is far stronger than that controlling specific sensitization to HDM allergens [Tovey et al., 1998]. Results were later confirmed for rye grass pollen [Sluyter et al., 1998] and *Alternaria* [Karihaloo et al., 2002]. These findings were corroborated in Danish adult twins in whom serum total IgE and positive SPT to at least one of ten aeroallergens were both shown to have a high heritability (81% and 54%, respectively) unlike HDM sensitivity of which genetic factors explained only 6% of the susceptibility (Table 3) [III]. Also, among Australian twins the heritability of HDM sensitivity was low (22%) compared with overall atopy (49%) [Ferreira et al., 2006].

Taken together, twin studies provide strong evidence for genetic determination of atopy but limited evidence to support inheritance of specific allergies. However, in a study among 826 randomly selected Chinese adolescent and adult twin pairs, the heritability of several specific sensitizations: HDM (66%), cockroach (64%), shellfish (54%), and peanut (51%), was high and comparable in magnitude to overall atopy (68%) [Liu et al., 2009]. In addition, a study of 58 twin pairs from the United States found that the heritability of peanut allergy was substantial (81%) [Sicherer et al., 2000]. It would be interesting to study in greater detail in twins when and to which allergens sensitization develops. This would require repeated measurements of skin test reactivity or RAST in large random twin series of children followed from birth.

Several other intermediate asthma phenotypes have been studied in twins, particularly airway responsiveness. Hopp and colleagues found that heritability explained 66% of the variance in methacholine reactivity [Hopp et al., 1984]. In Danish adult twins the heritability of airway responsiveness to methacholine, dose response slope (DRS) was 43% (Table 3) [III]. This is in accordance with reported estimates from Australia (58%) [Ferreira et al., 2006] and the Netherlands (47%) [Wu et al., 2010]. In contrast, a study of Norwegian adult twins (171 pairs) found that most of the variation in DRS was mediated by shared environmental effects and not by genetic effects [Lund et al., 2007]. The heritability of FeNO has been estimated to be 67% in Danish twins (Table 3) [III], 60% in Norwegian twins [Lund et al., 2007], and 58% in a small sample of Hungarian, Italian, and American twins [Tarnoki et al., 2013b]. The heritability of blood eosinophil count has been estimated to be 28% and 69% in two different samples of Australian twins [Evans et al., 1999; Ferreira et al., 2006], 52% in Dutch twins [Wu et al., 2010], and 64% in UK twins [Hall et al., 2000]. Interestingly, the CD4+/CD8+ ratio of T cells in peripheral blood is more correlated in MZ twins than in DZ twins [Yokoyama & Akiyama, 1995; Evans et al., 1999; Hall et al., 2000], and between 65% and 84% of the variation in CD4+/CD8+ ratio is explained by genetic factors [Evans et al., 1999; Hall et al., 2000], indicating a substantial influence of genetics on this trait with an important role in the atopic lymphocyte switch in asthma.

A few twin studies have examined the genetic and environmental contributions to the *association* between intermediate asthma phenotypes. With the exception of FEV_1_ and FVC that were highly genetically correlated (0.90), most intermediate asthma phenotypes correlated weakly regarding underlying genetic effects among Danish adult twins (Table 2) [III]. Notably, genetic effects on lung function indices (FEV_1_, FVC, and FEV_1_/FVC) and airway responsiveness (DRS) correlated weakly with markers of atopy (serum total IgE, positive SPT, and HDM sensitivity). There was some genetic overlap between airway inflammation (FeNO), and several atopic markers, with a genetic correlation between FeNO and serum total IgE of 0.37, between FeNO and positive SPT of 0.41, and between FeNO and HDM sensitivity of 0.44. However, in general the different traits were either weakly correlated or their association was explained mainly by environmental effects. This finding is in accordance with a large study of Australian twins, which used a similar method to determine the degree of genetic and environmental overlap between traits [Ferreira et al., 2006]. These studies support the hypothesis that although a proportion of genetic factors is shared between intermediate asthma phenotypes, it is mainly specific genetic pathways that regulate the expression of each trait individually. The association between intermediate asthma phenotypes was also studied in Dutch twins [Wu et al., 2010], in whom results indicated a stronger genetic relationship between the traits in comparison with the Danish [III] and the Australian [Ferreira et al., 2006] studies, but these differences between studies could be partly explained by small sample size and differences in the statistical methods used. Another study of Australian twins that included only children and adolescents (381 pairs) indicated a shared genetic liability for positive SPT and airway hyperresponsiveness to hypertonic saline [Clarke et al., 2000].

In Danish adult twins, DRS, FEV_1_ and FVC had significant genetic correlations with several clinical asthma symptoms, particularly wheezing and shortness of breath. Conversely, genetic effects on FeNO, serum total IgE, positive SPT, and HDM sensitivity did not overlap significantly with any of these clinical asthma symptoms studied (Table 2) [III]. This indicates a shared genetic liability between specific asthma symptoms, and airway hyperresponsiveness and decline in lung function, but not between asthma symptoms and IgE production [III]. This is consistent with a large GWA study of asthma, which found that most of the identified susceptibility loci for asthma were not associated with IgE [Moffatt et al., 2010].

Heterogeneity between reported heritability estimates of intermediate asthma phenotypes and between genetic correlations between these traits may be explained by diagnostic differences, differences in the demographics of the studied populations, and the choice of statistical software and model, e.g. whether the full or the most parsimonious variance components model was reported. Furthermore, a general problem of clinical twin studies is small sample size, which biases estimates of heritability upwards. In particular, almost none of the clinical twin studies performed document an effect of shared environment on various intermediate asthma phenotypes, although reported correlations between MZ and DZ twins for these traits indicate that such effects could be expected. Another bias may arise from non-random selection of twins for clinical studies. For example concordance measures for atopy have been shown to differ depending on whether proband selection or random selection was performed; and non-random selection of twins may bias results towards a higher degree of concordance, particularly among MZ twin pairs [Lykken et al., 1978]. In Danish adult twins two different ways of statistical adjustment for proband-ascertainment of twins resulted in different heritability estimates of positive SPT, and in very different genetic correlations between positive SPT and asthma [III; Thomsen et al., 2006]. Further, analysing airway responsiveness to methacholine as a continuous variable [III, Thomsen et al., 2009] resulted in a different heritability compared with when a threshold model was employed [Thomsen et al., 2006]. This suggests that the exact heritability estimate, because of its nature, being a fraction of variance, depends on the way the trait is measured, i.e. how much variation is present in the operationalisation. Such issues may lead to very different conclusions about the importance of genetic factors in asthma.

In conclusion, asthma is a complex disease characterized by a set of genetically heterogeneous intermediate phenotypes. Understanding the aetiology and functioning of these intermediate phenotypes is essential as they hold considerable promise for advancing personalised medicine, for differentiating asthma phenotypes, and for predicting and monitoring treatment response [Szefler et al., 2012]. As new endophenotypes for asthma are discovered, twin studies provide a first effort in determining the contribution of genetic and environmental factors to these traits.

## The atopic march

The sequential development of the atopic diseases is referred to as the *atopic march*, characterised by the progression of atopic dermatitis to asthma and allergic rhinitis during the first years of life [Spergel, 2010]. Individuals with atopic dermatitis have an increased risk of developing asthma and hay fever, both in childhood [van der Hulst et al., 2007; Håkansson et al., 2007] and later in life [Thomsen et al., 2005]. Specifically, their lifetime risk of asthma is about 40% [Wüthrich, 1999] and probably dose-dependent so that individuals with early-onset atopic dermatitis or more severe eczema have an even higher risk of later development of asthma [Lowe et al., 2008]. Furthermore, as many as 80% of individuals with asthma, notably atopic asthma, have hay fever [Knudsen et al., 2009]. While these relationships are well documented, controversy remains as to whether the atopic diseases are causally related or whether they are diverse clinical manifestations of a common underlying (genetic) disease trait.

The causes for development of asthma and allergic rhinitis in the context of atopic dermatitis are imperfectly understood. However, recent discoveries have led to the formulation of a *leaky barrier hypothesis* stating that the skin acts as the site of primary sensitization through defects in the epidermal barrier with secondary reactivity in the airways [Spergel, 2010]. Compiling evidence centres on inherited defects in *filaggrin* [Kubo et al., 2012] but possibly also in other epidermal proteins [Walley et al., 2001; Oji et al., 2010] as the initiating event of the atopic march. Filaggrin, encoded by the epidermal differentiation gene cluster on chromosome 1q21, aggregates keratin filaments, flattens corneocytes, assists lamellar body loading and meshes with lipids liberated from the terminally differentiated corneocytes resulting in the cornified epidermal envelope, which is critical for skin barrier function [Burgess et al., 2009]. Deficient filaggrin leads to epidermal defects, increased transepidermal water loss, and possibly to increased penetration of antigens into the skin, allowing skin-resident antigen-presenting cells such as Langerhans or dendritic cells to capture environmental antigens [De Benedetto et al., 2012]. In addition, barrier-disrupted keratinocytes release immune adjuvants that activate and mature these innate immune cells as well as affecting their ability to direct naive T cell polarisation, thereby affecting the character of the T cell response [De Benedetto et al., 2012]. Filaggrin is expressed in the cornified epithelium of the skin, the oral mucosa, and the nasal vestibule, but apparently *not* in the bronchial or the gastrointestinal epithelium [De Benedetto et al., 2008]. Therefore, loss-of-function mutations in the filaggrin gene (*FLG*) are unlikely to directly affect barrier function and allergen reactivity in the lungs or other distant target organs. Instead, filaggrin-deficiency driven primary percutaneous allergic sensitization is speculated to lead secondarily to hyperactive airways and allergic airways disease [De Benedetto et al., 2008; Kubo et al., 2012] and possibly also to other atopic manifestations such as food allergy [Brown et al., 2011] and eosinophilic oesophagitis [Blanchard, et al., 2010].

*FLG* loss-of-function mutations were first coined as causative variants in the cornification disorder *ichthyosis vulgaris*, which is also a common clinical phenomenon in patients with atopic dermatitis [Smith et al., 2006]. *FLG* mutations are present in little under 10% of the European population but in as many as half of all patients with atopic dermatitis [Palmer et al., 2006]. Several studies have associated *FLG* loss-of-function mutations with atopic dermatitis. A systematic review and meta-analysis of genetic epidemiological studies showed that the risk of atopic dermatitis among individuals with *FLG* defects was increased about two times in family studies and almost five times in case-control studies [van den Oord & Sheikh, 2009]. Moreover, the risk of allergic sensitization and allergic rhinitis was also increased, as was the risk of asthma but *only* in those with coexistent atopic dermatitis. These findings provide strong supporting evidence that, at least in a subset of those with atopic disease, *FLG* defects may be the fundamental predisposing factor not only for the development of eczema but also for initial sensitization and progression of allergic disease [van den Oord & Sheikh, 2009]. Of particular note is the association between *FLG* defects and asthma selectively in patients with the coexistence of atopic dermatitis supporting the hypothesis that asthma is secondary to allergic sensitization occurring after skin barrier disruption [Spergel, 2010]. Recently, however, an association has been reported between *FLG* loss-of-function mutations and asthma independently of atopic dermatitis in a Polish [Poninska et al., 2011] and a Chinese [Li et al., 2011] population, respectively. The implications of these findings are currently unclear.

Twin studies have shown that most of the association between the atopic diseases can be explained by a shared genetic liability. This is exemplified by the observation that MZ twins are often more concordant for different pairs of atopic diseases (e.g. atopic dermatitis and asthma) than are DZ twins. (Table 4). Specifically, in a large sample of Danish twins 12–41 years of age, as much as 81% of the phenotypic relationship between atopic dermatitis and asthma was mediated through pleiotropic genetic effects, whereas 85% of the relationship between atopic dermatitis and hay fever, and 70% of the relationship between asthma and hay fever, was ascribable to such common genetic effects [IV]. These findings are consistent across age groups and countries [Duffy et al., 1990; Lichtenstein & Svartengren, 1997; van Beijsterveldt & Boomsma, 2007; Willemsen et al., 2008; Fagnani et al., 2008]. A direct interpretation of this is that the susceptibility to the different atopic diseases is largely determined by a common set of genetic factors and to a lesser extent also by disease-specific or disease-modulating genetic factors. This lends support to the hypothesis of a common (genetic) underlying atopic disease trait of which atopic dermatitis, asthma, and hay fever, respectively, are causally independent but sequentially occurring manifestations.

**Table 4:** Risk of atopic diseases in a sample of 29,183 Danish adolescent and young adult twins

	Asthma	Hay fever	Atopic dermatitis
MZ twins			
Asthma	20.69 (15.08–28.38)		
Hay fever	4.30 (3.20–5.56)	14.28 (11.40–17.90)	
Atopic dermatitis	3.81 (2.72–5.32)	2.87 (2.17–3.78)	32.98 (24.66–44.11)
DZ twins			
Asthma	4.24 (2.97–6.06)		
Hay fever	2.00 (1.46–2.73)	3.10 (2.49–3.85)	
Atopic dermatitis	1.73 (1.16–2.58)	1.40 (1.02–1.93)	5.63 (4.20–7.56)

Odds ratios (95% confidence intervals) denote the risk of an atopic disease in a co-twin of an affected twin relative to a co-twin of an unaffected twin. Rows are diseases in the index twin and columns are diseases in the co-twin. Modified from [IV].

Because these different diseases share common systemic characteristics, it is reasonable to propose that a number of susceptibility genes contribute to the allergic process regardless of the specific clinical phenotype [Barnes, 2000]. However, molecular genetic studies have shown a high degree of genetic heterogeneity within the atopic phenotype. For example, separate GWA studies, respectively, of asthma [Moffatt et al., 2010] and atopic dermatitis [Paternoster et al., 2011; Sun et al., 2011] found no regions common to the two diseases. Further, a GWA study found no evidence of interaction between hay fever and loci contributing to asthma [Ramasamy et al., 2012]. Although it is difficult to translate relative genetic effects into actual genes, these findings of molecular studies could indicate that the high genetic similarity between the atopic diseases that has been found in twin studies possibly reflects acquired characteristics of the atopic syndrome rather than shared genes. This would also help explain why many children do not complete the atopic march, i.e. why some develop only atopic dermatitis and not asthma, and contrary to this, why many develop asthma without pre-existent atopic dermatitis.

Environmental effects quite possibly also play a role in the differentiation into a specific atopic trajectory. In support of the evidence provided by twin studies, population studies of singletons point to differential environmental risk profiles for asthma, hay fever, and atopic dermatitis. For example, exposure to passive smoking in childhood or in utero is a well established risk factor for asthma [Magnusson et al., 2005; Lee et al., 2012] but seems not to affect, or may even lower, the risk of atopic dermatitis and hay fever [Strachan & Cook, 1998; Magnusson et al., 2005; Lee et al., 2012]. Another example is socioeconomic status (SES), which seems to have a differential effect on the risk of asthma [Gold & Wright, 2005] and atopic dermatitis [Weber & Haidinger, 2010; Shaw et al., 2011], with asthma occurring predominantly in families with low SES, and atopic dermatitis occurring more often in families with high SES. Furthermore, birth anthropometric factors have been coupled differentially to asthma and atopic dermatitis; decreased fetal growth is a risk factor for asthma [Turner, 2012] but seems to have the opposite effect on the risk of atopic dermatitis [Lundholm et al., 2010]. A study of multiple risk factors for atopic dermatitis and infant wheeze found differential and essentially opposing effects of various risk factors for these diseases pertaining to sex, maternal age, maternal occupation, smoking during pregnancy, season of birth, birth weight, gestational age, head circumference, breast-feeding, number of older siblings, day-care attendance, and pets in the home [Linneberg et al., 2006]. Although infant wheeze is often transient and different from persistent asthma in childhood [Martinez et al., 1995; Spycher et al., 2008], these results still support a different environmental aetiology for asthma and atopic dermatitis.

The chain of events that links asthma to atopic dermatitis and hay fever is complex and involves a multitude of hereditary and developmental factors that exert their effect in the context of environmental exposures. Emerging data indicate a causal link between atopic dermatitis and asthma and hay fever mediated by an innate deficient skin barrier. Notably, it is becoming evident that the process of allergic sensitization and the progression of atopic dermatitis to asthma and allergic rhinitis arise from the dynamic crosstalk between a deficient skin barrier and the immune system [De Benedetto et al., 2012]. However, this course may be relevant only for certain types of asthma, particularly classical atopic asthma with early onset, whereas adult-onset asthma or non-atopic asthma may result from different pathways. Moreover, it is not clear whether the co-occurrence of asthma and hay fever in the absence of pre-existing atopic dermatitis constitutes an exception to this.

Previously, twin studies of the atopic triad have been based mainly on retrospective questionnaire data, whereas no clinical, prospective twin studies address the progression of atopic dermatitis to asthma. Such studies would preferably include candidate genetic marker data, such as *FLG* variants, as well as detailed clinical data on the onset and severity of atopic diseases. It would be interesting to examine how much variance of asthma is attributable to *FLG* variants in individuals with pre-existing atopic dermatitis compared with individuals with sporadic asthma. Furthermore, clinical trials of patients with atopic dermatitis or genetically mediated skin barrier defects that intervene on skin barrier function would be valuable, as an observed reduced risk of asthma following intervention would signal the possibility of primary prevention of asthma [Simpson et al., 2010]. The intricate interplay between structural epidermal proteins, immune mechanisms, and local homeostatic factors involved in skin barrier function remains to be elucidated. This could hold the key to understanding the process of sensitization and progression of allergic disease. In this regard, candidate gene studies relating to factors other than filaggrin involved in skin barrier function would help explain why less than half of all patients with atopic dermatitis develop asthma and, conversely, why many without atopic dermatitis and *FLG* defects still develop asthma. Further, filaggrin *counterparts* may exist in the airways and gut mucosal linings, for example *E-cadherin* [Nawijn et al., 2011], and search for genetic defects leading to deficiencies in such structural epithelial proteins may help solve this enigma.

## The hygiene hypothesis

In a UK population study from 1989, Strachan observed that birth order and family size were inversely related to development of hay fever and eczema [Strachan, 1989]. This observation prompted the *hygiene hypothesis*, which speculates that a decreased exposure to infections along with a concurrent increase in the use of antibiotics and a resulting *cleaner* environment in Western societies over the past decades has led to a higher prevalence of atopic diseases in the population. Notably, declining family size, improvements in household amenities, and higher standards of personal cleanliness have reduced the opportunity for cross infection, which may have resulted in more widespread clinical expression of atopic diseases via a deviation towards T_H_2 immune activity.

Pregnancy is a state of relative T_H_2 dominance and babies tend to be born with T_H_2-biased immune responses. These can be switched off rapidly postnatally under the influence of microbial exposure or can be enhanced by early exposure to allergens [Berger, 2000]. From an evolutionary perspective, T_H_2 immune responses are thought to have evolved to resist infection by parasites, particularly helminths. In contrast, T_H_1 responses have important roles in killing intracellular pathogens and in perpetuating autoimmune responses [Berger, 2000]. Modern human's ancestors lived in an environment where infectious, tropical diseases would have been endemic, causing genetic selection for increased T_H_2 proinflammatory immune responses. On migrating to temperate regions, pronounced proinflammatory responses would have been less important and selected against due to increased mortality from overly vigorous responses to harmless environmental agents. Consequently, the reduction in the risk from parasites was counterbalanced by an increased inherited propensity to atopic diseases [Le Souëf et al., 2000; Le Souëf et al., 2006].

### Secular trends in the occurrence of asthma

During the second half of the last century, the occurrence of asthma and other atopic diseases increased considerably worldwide. Changes in lifestyle and environment, so-called *Westernisation*, have been postulated as the primary cause for this, mainly since the rising incidence of atopic diseases has occurred more rapidly than changes to the genome sequence would allow [Douwes & Pearce, 2002; Bach, 2005]. Schnyder cites several population studies of asthma, many from the first half of the 19^th^ century [Schnyder, 1960]. Although some of these studies report a prevalence of asthma between 2% and 7%, the presented evidence is consistent with a prevalence of below 1% in the European population before the 1960s. During the past decades asthma has risen to epidemic proportions in many countries, particularly in Western societies but also in developing countries. The reasons for this are imperfectly understood but some have pointed to an increased recognition of asthma and to differences in the way we currently diagnose the disease compared with how it used to be diagnosed. However, carefully conducted serial cross-sectional population studies using similar diagnostic methods of asthma on two occasions have documented that asthma has increased in prevalence, particular since the 1960s [Anderson et al., 2007]. In Denmark there has been an increase in the prevalence of asthma among both children [Thomsen et al., 2004] and adults [Linneberg et al., 2001] during those years.

Recent studies indicate that the prevalence of asthma has now reached a plateau or in some instances has even declined in countries with a formerly high incidence. For example, the International Study of Asthma and Allergies in Childhood (ISAAC) compared worldwide asthma prevalence rates in school children between 1994 and 2003 and found an increase in the prevalence of asthma in about 40% of the countries studied [Asher et al., 2006]. However, countries with a formerly high prevalence of asthma, particularly from Western Europe, tended to experience a decrease in prevalence, whereas the prevalence of asthma in several developing countries was still on the increase.

Clues to the causes of this widespread increase in asthma prevalence come from studies of migrants. More specifically, immigrants to the industrialized world from the developing world increasingly develop allergic disorders in relation to the length of time since arrival in the industrialized world. For example the prevalence rates of asthma and allergic diseases among immigrants from South-East Asian countries increase with the duration of residence in Australia so that after ten years in Australia, up to 60% of South-East Asian immigrants have developed hay fever while 15% have symptoms of asthma [Leung, 1996]. Furthermore, studies of *indigenous* populations show a protective effect of *traditional* lifestyle on the risk of asthma explained by gene-environment interaction, for example Greenlander Inuits residing in Greenland have a lower risk of asthma compared with Greenlander Inuits who have moved to Denmark [Candelaria et al., 2010].

Contrasting epidemiological trends are also observed locally. For example, the prevalence of asthma was significantly higher in West Germany compared with East Germany shortly after their reunification, suggesting an impact of differential environments on two ethnically similar populations [von Mutius et al., 1994]. Only a few years later, prevalence rates had converged as an indication that more congruent lifestyles had developed [Heinrich et al., 2002]. Also, the frequency of allergic diseases is different in Finnish and Russian Karelia, two neighbouring geographical regions with the same ethnic background: Finland has a fivefold higher allergy incidence [Laatikainen et al., 2011], suggesting differential genetic expression as a result of environmental variation [Zhang et al., 2009; Zhang et al., 2011].

**Fig. 5:**
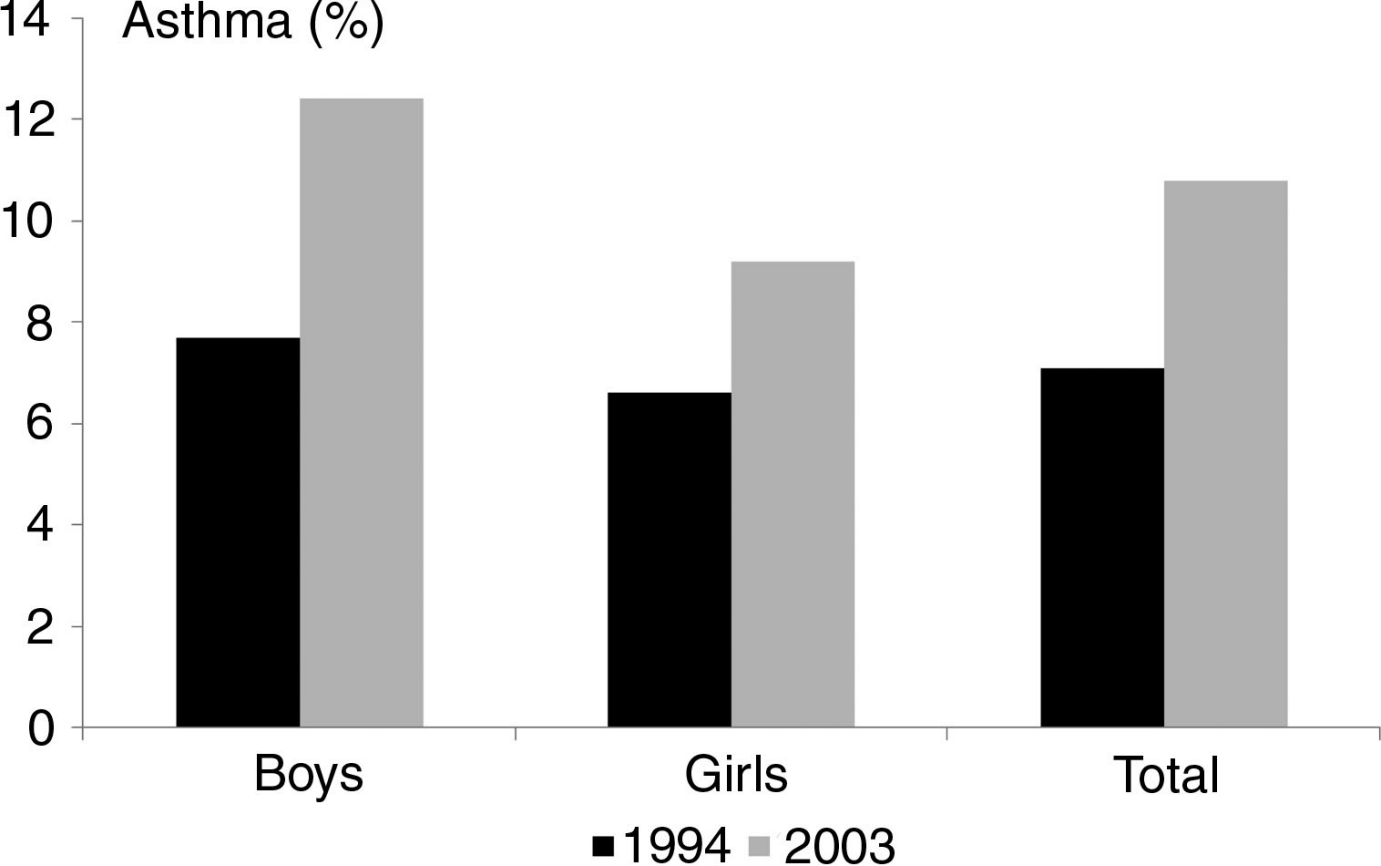
Change in prevalence of asthma between 1994 and 2003 among Danish adolescent twins.

These results point to a genetic sensitivity to widespread environmental changes in the studied populations leading to an increased occurrence of asthma over time. A Danish twin study has provided evidence in favour of this hypothesis being the first to study changes in prevalence and heritability of asthma over time [V]. In this study of Danish adolescent twins, the prevalence of self-reported asthma increased from 7.1 to 10.8% between 1994 and 2003 [V]. The increase in prevalence was observed both among boys and girls (Figure 5). In the same period the heritability of asthma increased significantly from 79 to 91%. This was particularly due to an increased concordance for asthma among MZ twins in 2003 compared with 1994 (0.73 vs. 0.50), whereas the concordance for asthma among DZ twins was more or less unchanged between 2003 and 1994 (0.29 vs. 0.24); the ratio between concordance rates in MZ and DZ twins increased from 2.08 to 2.52 during these years. Although this result could be due to a decrease in the overall variance in the asthma question used on the two occasions, the data fit well with the hypothesis that the prevalence of asthma has increased globally due to widespread environmental changes. Notably, the influence of genetic factors seems to have increased over time as a result of environmental changes. That is, the extent to which genetic influences affect asthma has increased as a reaction to these environmental changes leading to a higher heritability of asthma in the more recent generations [V].

Intriguingly, the hygiene hypothesis has been extended to include inflammatory diseases in general, particularly several *T*_*H*_*1-mediated* autoimmune diseases, such as multiple sclerosis, inflammatory bowel disease and type 1 diabetes [Bach, 2002], and also certain lymphomas [Bach & Chatenoud, 2012]. These diseases show remarkably similar geographical distributions and epidemiological patterns compared with the atopic diseases [Bach, 2002; Bach, 2005]. A low prevalence of autoimmune diseases is chiefly observed in the tropical regions, where infections are prominent, whereas in more temperate regions their occurrence is high [Bach & Chatenoud, 2012]. Interestingly, as atopic diseases and autoimmune diseases seem to follow similar epidemiological trends, they are expect to be inversely related in the individual. Notably, while atopic diseases are dominated by production of T_H_2 cytokines, such as IL-4, IL-5, and IL-13, autoimmune diseases, such as type 1 diabetes, are dominated by the T_H_1 cytokines IL-2 and interferon gamma (IFN-γ). However, this T_H_1/T_H_2 dichotomy represents a simplified view of the immunological mechanisms underlying these diseases. For example, in *chronic* asthma - unlike in acute asthma - T_H_1 cytokines have also been shown to play a prominent role [Barnes, 2008], whereas in type 1 diabetes, T_H_2 mechanisms are important [Azar et al., 1999]. Furthermore, other immune cells, such as regulatory T cells and T_H_17 cells and their respective cytokines, as well as aspects of the innate immune system, have similarly been shown to play important roles in the pathogenesis both of asthma [Barnes, 2008] and autoimmune diseases, such as type 1 diabetes [Kim & Lee, 2009].

**Fig. 6:**
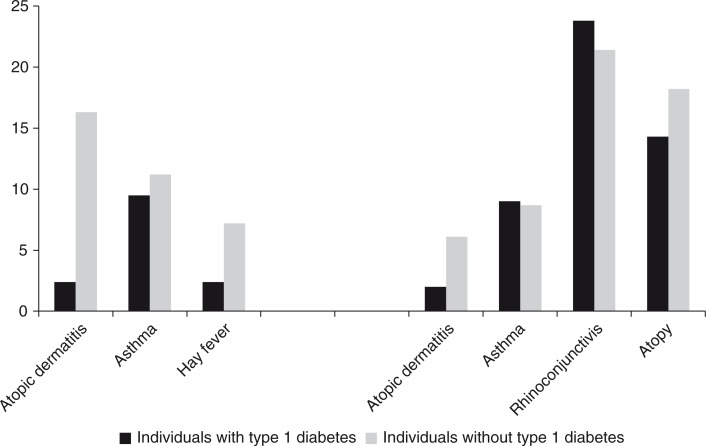
Risk of atopic diseases in Danish twins with and without type 1 diabetes. Note: Prevalence (%) of atopic diseases in child and adolescent twins (left panel) and adult twins (right panel).

An inverse association between atopic diseases and type 1 diabetes has been found in several, albeit not all [Stene & Nafstad, 2001], observational studies of singleton populations, both in relation to asthma, hay fever, and atopic dermatitis and also in relation to allergic sensitization [Cardwell et al., 2003]. However, only one twin study has examined this [VI]: in Danish child and adolescent twins with hospital diagnosed type 1 diabetes, the risk of self-reported asthma was found to be slightly, but not statistically significantly, lower compared with twins without type 1 diabetes (9.5 vs. 11.2%), whereas the risk of asthma in adult twins was about the same in subjects with and without type 1 diabetes, respectively (9.0 vs. 8.7%), supporting that asthma *in children* may be inversely related to type 1 diabetes (Figure 6) [VI]. A composite measure of any self-reported atopic disease, i.e. asthma, hay fever and/or atopic dermatitis, was significantly less prevalent in child and adolescent twins with type 1 diabetes compared with non-diabetic individuals (11.9 vs. 28.0%). This effect was driven primarily by a strong inverse relationship between atopic dermatitis and type 1 diabetes, which was present both in children and adults; the prevalence of atopic dermatitis in individuals with and without type 1 diabetes was 2.1% and 9.9%, respectively. Of particular note was a significant negative genetic correlation between type 1 diabetes and atopic dermatitis of −0.30 and a substantial positive, albeit not statistically significant, non-shared environmental correlation of 0.52, indicating that atopic dermatitis and type 1 diabetes are regulated partly by opposing genetic mechanisms but, in contrast, seem to share environmental risk factors to a sizable extent [VI]. This observation seems to fit well with the hygiene hypothesis as a coherent explanation for the recent increase in the prevalence both of atopic diseases and autoimmune diseases that also accommodates the contrasting risk for these diseases within the individual (the T_H_1/T_H_2 paradigm). Specifically, the observed concomitant decrease in the incidence of many infectious diseases and the resulting change in the quality of the microbial burden because of the recent improvements in hygiene, use of antibiotics, vaccinations and better socioeconomic conditions in developed countries in the last half of the 20^th^ century seems to have led to an increase in the prevalence of autoimmune diseases as well as atopic diseases at the population level while at the same time conserving an inverse association between them on the individual level.

### Role of microbial stimulation in the aetiology of asthma

Observational studies have coupled the recent increase in the prevalence of asthma and atopic diseases in Western societies to the general decline in the occurrence of several *prototypical* infections such as *tuberculosis*, *measles*, *hepatitis A*, and *helicobacter pylori* [Bach, 2002]. Evidence stems primarily from studies of infectious exposures that theoretically or evidentially promote a T_H_1 immune response and/or mitigate a T_H_2 response. For example, mycobacterial infections as well as immunization with Bacille Calmette-Guérin (BCG) favour a lymphocyte switch towards a non-atopic cytokine profile and thereby induce a T_H_1 immune response [Marchant et al., 1999]. Some studies [Shirakawa et al., 1997; El-Zein et al., 2010; Arnoldussen et al., 2011], but not all [Flohr et al., 2012], have shown that BCG vaccination confers a protective effect on the development of asthma. In an observational study of Danish twins, being BCG vaccinated decreased the risk of asthma by about 15% [II]. However, data were retrospective and based on self-reported exposure and outcome, and an attempt to verify the protective effect of BCG vaccination on asthma development in twin pairs discordant for BCG vaccination did not show a protective effect in the immunized twin [Thomsen et al., 2008a]. Likewise, measles and pertussis infection has been found to confer a protective effect on childhood asthma but evidence is contradictory [Shaheen et al., 1996; Nagel et al., 2012], particularly from experimental trials [Nilsson et al., 1998]. Furthermore, seropositivity to hepatitis A has been inversely associated with atopic diseases [Matricardi et al., 1997; Linneberg et al., 2003], but, similarly, evidence is circumstantial [Veiga et al., 2011]. Interestingly, subjects expressing the long form of the hepatitis A virus TIM-1 receptor on T_H_2 cells have a lower frequency of atopic diseases and a higher susceptibility to severe forms of hepatitis A [Bach & Chatenoud, 2012]. Helminthic infestations, particularly with *hookworm*, have also been shown to confer protection against asthma and allergic diseases [Leonardi-Bee et al., 2006]. Notably, allergic diseases are rare in areas with high helminth parasite exposure and common where helminth exposure is lacking or significantly reduced, such as urban areas of developing countries and industrialized countries. Helminths are thought to induce a systemic immuno-modulatory network, including regulatory T cells and anti-inflammatory IL-10, which might play a key role in the protection against the allergy [Flohr et al., 2009]. Finally, several studies have associated infection with Helicobacter pylori to a decreased risk of asthma, a possible mechanism being gastric recruitment of regulatory T cells [Blaser, 2012].

Further insight into the mechanisms responsible for the protective effect of microbial stimulation on the risk of asthma stems from studies of rural populations and populations with *anthroposophic* background. Notably, children brought up in a *traditional* farming environment [Genuneit, 2012] or in families with an anthroposophic lifestyle [Alm et al., 1999; Flöistrup et al., 2006] have a lower risk of atopic diseases compared with children from urban dwellings. Studies performed in Central Europe (Germany, Austria, and Switzerland) and also in other countries, suggest that protection is mostly related to dairy farming as opposed to other types of farming [von Mutius, 2012]. The reasons for this have been speculated to be exposure to high levels of bacterial endotoxin from livestock [Braun-Fahrländer et al., 2002] but also to constituents of unpasteurized cow's milk [von Mutius, 2012]. Particularly, ingestion of components of unprocessed cow's milk, such as microorganisms and heat labile components of the whey fraction, may help create an intestinal microenvironment that promotes a non-atopic immune switch [van Neerven et al., 2012]. Further, farm exposure has been shown to influence epigenetic and expression patterns throughout a range of asthma candidate genes including innate immunity genes such as Toll-like receptor genes [Loss et al., 2012] and genes contributing to T cell differentiation into T_H_2 and regulatory T cells [Michel et al., 2013].

Approaching the 25^th^ anniversary of the initial formulation of the hygiene hypothesis, the *disappearing microbiota (microflora) hypothesis* now acts as a more general paradigm explaining the diseases of modernity including atopic diseases and other inflammatory diseases. Specifically, humans and our ancestors have evolved since the most ancient times with a *commensal* microbiota [Blaser & Falkow, 2009]. The microbiota aids in the digestion of foods and nutrient absorption, protects against colonization by pathogens, degrades mucin, and promotes the differentiation of epithelial cells and mucosal-associated lymphoid tissue [Frei et al., 2012]. In addition, the composition and metabolic activity of the microbiota have profound effects on the induction of immune tolerance. Notably, colonisation, expansion, and maturation of gut microbial populations during infancy coincide with a switch from a fetal T_H_2-dominated to a mature T_H_1-dominated immune profile [Cozen et al., 2013]. Thus, the activity of the human immune system seems to be governed by the balance between symbiotic and pathogenic factors derived from our microbial inhabitants [Frei et al., 2012].

Recent advances in genome sequencing techniques have allowed a more detailed investigation of the genome sequence of these microorganisms (the human *microbiome*) and have consequently led to a more profound characterisation of the microbial colonization of the human barrier organs. Interestingly, pronounced differences in bacterial assemblages and functional gene repertoires exist between populations from different countries and these distinctive features are evident already in early infancy [Yatsunenko et al., 2012]. Notably, MZ twins and their mothers share a significantly greater degree of similarity in their faecal microbial communities than do unrelated individuals, suggesting that host genetic factors contribute to the variation in the innate microbial colonization of humans [Reyes et al., 2010]. However, although the human gut microbiome is shared among family members, each person's gut microbial community varies in the specific bacterial lineages present, with a comparable degree of co-variation between MZ and DZ twin pairs [Turnbaugh et al., 2009].

It is becoming increasingly evident that *dysbiotic* disturbances to the commensal microflora of the human barrier surfaces, i.e. gut, skin, vagina, and airways, probably constitute a fundamental causative insult in asthma. Particularly, over-expression of specific microbial species in these body habitats at the expense of microbial diversity may play a causative role in asthma. Several studies have demonstrated that the microbiota of allergic and non-allergic infants differs even before the development of symptoms, with a critical time window during the first six months of life [Vael et al., 2011]. Recent data suggest that farm exposure to endotoxin may be an indicator of exposure to a multitude of microbial organisms, rather than being causative itself, and consequently that *reduction* in microbial diversity is associated with asthma development [Ege et al., 2011]. In a study from Central Europe, children growing up on farms were exposed to a greater variety of environmental fungi and bacteria than were children in a reference group who lived in the same regions. The greater diversity of environmental microbial exposure was inversely related to asthma, independently of farming, supporting the idea that the greater diversity of microbial exposure among children who lived on farms was associated with protection from asthma development [Ege et al., 2011]. A study of Danish non-twin children with atopic predisposition showed that decreased diversity of species in the infant intestinal flora was associated with an increased risk of sensitization and allergic rhinitis, but not asthma, at school age, supporting the idea that reduction in the diversity of the commensal microbiota could play a causative role in atopic diseases [Bisgaard et al., 2011]. Similar findings were observed among Swedish children in relation to atopic dermatitis [Abrahamsson et al., 2012], and among Flemish and Dutch children, respectively, in relation to asthma [Vael et al., 2011] and wheezing [Penders et al., 2007], whereas a study of different populations of European children did not find an association between infant intestinal microbial diversity and atopic dermatitis or sensitization [Adlerberth et al., 2007]. Of further interest, neonates colonized in the hypopharyngeal region with *Streptococcus pneumoniae, Haemophilus influenzae*, or *Moraxella catarrhalis*, or with a combination of these organisms, are at increased risk for subsequent recurrent wheezing and asthma, suggesting that colonization with certain bacterial taxa could be causative rather than indicative of asthma [Bisgaard et al., 2007]. A possible mechanism for this relationship is induction of specific immune responses, as colonization of the airways of asymptomatic neonates with Haemophilus influenzae or Moraxella catarrhalis is associated with a mixed T_H_1/T_H_2/T_H_17-type inflammatory immune response profile of the airway mucosa, which may result in chronic inflammation [Følsgaard et al., 2013].

A specific exposure that has been speculated to promote asthma development through altered microbial stimulation is Caesarean section delivery. Caesarean section delivery has been suspected to predispose to asthma via interference with lung physiology due to lack of thoracic decompression during delivery [Thavagnanam et al., 2008]. However, in congruence with the microflora hypothesis, vaginally delivered infants acquire bacterial communities resembling their own mother's vaginal microbiota, whereas infants delivered by Caesarean section harbour bacterial communities similar to those found on the skin surface [Dominguez-Bello et al., 2010]. Specifically, Caesarean section delivery has been shown to preclude perinatal exposure to maternal faecal and vaginal microbes, particularly *Bifidobacteria, Bacteroides, Lactobacillus, Prevotella*, and *Sneathia*, which have probiotic properties that may be associated with a decreased risk of atopic disease, and in contrast, promote colonization with harmful bacterial strains such as *Clostridium difficile, Staphylococcus, Corynebacterium*, and *Propionibacterium* [Adlerberth et al., 2007; Dominguez-Bello et al., 2010] with changes persisting for months postnatally [Grönlund et al., 1999]. Systematic reviews and meta-analyses of observational studies have shown that Caesarean section increases the risk of asthma by about 20% [Thavagnanam et al., 2008; Bager et al., 2008]. Interestingly, children born via elective Caesarean section seem to carry a small excess risk of later development of asthma compared with children born via acute Caesarean section [Bager et al., 2008]. Rupture of fetal membranes, while rare before elective Caesarean sections, is frequent before acute Caesarean sections, allowing ascending spread of vaginal bacteria to the fetus [Bager et al., 2008]. Apparently, this effect seems not to be counterbalanced by the excess risk of asthma due to neonatal respiratory distress associated with acute Caesarean section, supporting the notion that the harmful effect of Caesarean section alludes essentially to dysbiosis rather than, or in addition to, factors associated with fetal respiratory distress. In Danish twins, the risk of asthma in children born via Caesarean section was increased by as much as 75% [Kahr et al., 2013]. Notably, the heritability of asthma was found not to be significantly different in children born via Caesarean section compared with children delivered vaginally, i.e. Caesarean section did not modify the genetic influence on asthma, suggesting that Caesarean section may promote asthma development directly via an abnormal microbial colonization of the newborn. However, intriguingly, women whose first child was delivered via Caesarean section are more likely to have asthma themselves, and this could have overestimated the detrimental effect of this exposure [Jackson et al., 2012]. A harmful effect of Caesarean section in relation to asthma has also been observed in Dutch twins [van Beijsterveldt & Boomsma, 2008].

Several studies have shown that antibiotic use in early childhood increases the risk of later asthma, and also that antibiotic use by the mother during pregnancy increases the risk of asthma in the offspring [Murk et al., 2011]. However, most of these studies suffer from methodological weaknesses, and the results are suspected to be influenced by reverse causality or *protopathic* bias, which occurs when early symptoms of undiagnosed asthma are attributed mistakenly to respiratory infections and are treated with antibiotics [Murk et al., 2011]. However, a Danish study of non-twin children found an increased risk of asthma associated with maternal antibiotic use during pregnancy in a clinical birth cohort predisposed to asthma and replicated this finding in an unselected national birth cohort and in a subgroup using antibiotics for non-respiratory infections, thereby minimising these biases [Stensballe et al., 2013]. In congruence with the microflora hypothesis, antibiotic use has been shown to suppress commensal bacteria, such as Bifidobacteria and Bacteroides, and permit emergence of Clostridium difficile and other harmful bacterial strains [Penders et al., 2006] with disturbances persisting for years [Jernberg et al., 2007], and probably through these pathways leads to asthma [Russell et al., 2012].

The hygiene hypothesis provides a solid framework for understanding the asthma epidemic, but to date the exact mechanisms of interaction between environmental exposures and the immune system are imperfectly understood and there appear to be several inconsistencies. Uncertainty pertains to the often differential findings for asthma compared with the other atopic diseases and for the different subtypes of asthma (atopic and non-atopic) in relation to microbial exposure [Brooks et al., 2013]. Further, routes of microbial colonisation of neonates have not been fully elucidated. Moreover, increased knowledge about host-microbiota interplay has not translated into preventive measures. For example, trials of probiotics for primary prevention have shown some benefit in relation to atopic dermatitis [Pelucchi et al., 2012] but have been disappointing in relation to asthma [Osborn & Sinn, 2007]. Similarly, substitution with helminthic products has been thought of as a potential means of prevention but human intervention studies have provided only little support in favour of such measures [Flohr et al., 2009]. Well-designed future twin studies may help elucidate the complex interplay between genetic factors, microbial colonisation, and environmental exposures in the search for preventive strategies.

### Early life respiratory viral infections

Studies of infant bronchiolitis, particularly bronchiolitis caused by *respiratory syncytial virus* (RSV), have shown that respiratory viral infections encountered early in life constitute an exception to the general rule that microbial stimulation confers a protective effect on asthma development. RSV affects most children at some point during their first two years of life, and it has been estimated that at least 33.8 million episodes of RSV-associated acute lower respiratory infection occur worldwide per year in children younger than five years of age [Nair et al., 2010]. This corresponds to approximately 22% of all episodes of acute lower respiratory infection in young children [Nair et al., 2010]. The incidence of severe RSV infection in developing countries has been reported to be more than twice that in Western countries, in which the hospitalisation rate for RSV-bronchiolitis is 1–3% [Welliver, 2003].

It is not clear why only a small number of RSV-infected children develop severe respiratory disease requiring hospitalisation, whereas the majority have only mild disease. However, prematurity, T cell immunodeficiency, chronic lung disease, and congenital heart disease are established risk factors [Hull et al., 2000]. Genetic background also determines the clinical outcome of RSV infection. A Danish twin study of 3–9-year-old children found that genetic effects explained approximately 20% of the variation in the risk of RSV hospitalisation [Thomsen et al., 2008b]. In contrast, common environmental effects, highlighting the infectious nature of RSV, explained most of the remainder of the variation. Susceptibility to severe RSV infection has been associated with genetic polymorphisms in several immune-related genes such as the *IL-8* [Hull et al., 2000], *IL-10* [Wilson et al., 2005], *IL-13* [Puthothu et al., 2006], *RANTES* [Amanatidou et al., 2008], chemokine receptor *CX3CR1* [Amanatidou et al., 2006] and surfactant proteins *A* [Löfgren et al., 2002] and *D* [Lahti et al., 2002] genes, indicating that a deficient cytokine response is associated with risk of severe disease.

Severe RSV infection in infancy is a firmly established risk factor for subsequent asthma, wheezing, and abnormal pulmonary function in later childhood [Singh et al., 2007]. Notably, a Swedish study found a risk of asthma of 43% among 13-year-old children with a history of early RSV bronchiolitis compared with only 8% in a matched control group [Sigurs et al., 2005]. Furthermore, a study from the United States reported an over four-fold increased risk of frequent wheeze in six-year-old children who had had severe RSV-induced lower respiratory tract illness in infancy compared with children without infant lower respiratory tract illness [Stein et al., 1999]. The risk was markedly higher than among those children who had had other viral infectious aetiologies such as *para-influenza virus*. Moreover, the increased risk was not explained by an increased rate of atopic sensitization, and was no longer increased at age 13 years. These results support that almost half of all children with a history of hospitalisation for RSV bronchiolitis go on to develop asthma at some point later in childhood. The results are also in line with the findings of a systematic review and meta-analysis of observational studies comprising ~82,000 individuals, which showed that children who had had severe RSV disease in early life have an approximately four-fold higher incidence of asthma/wheezing in later childhood, but with a decrease in risk with age at follow-up [Régnier & Huels, 2013].

Contrary to these findings, several studies have found that predisposition to asthma increases the risk of lower respiratory tract infection and RSV hospitalisation. For example, early wheezy symptoms were found to be a strong risk factor for subsequent RSV hospitalisation [Stensballe et al., 2006], whereas impaired pulmonary function [Turner et al., 2002] and airway hyperresponsiveness [Chawes et al., 2012] measured at one month of age increased the risk of acute severe bronchiolitis in response to infections with respiratory tract viruses, particularly RSV, in later childhood. Finally, several of the genetic variants associated with severe RSV infection have also been implicated in the susceptibility to asthma [Singh et al., 2007].

While the strength of the association between severe RSV infection and asthma is well described, the nature of this association remains imperfectly understood. It is unclear whether severe infant RSV infection plays a direct causative role in asthma or simply unmasks a genetic predisposition for subsequent asthma, wheezing, and reduced pulmonary function later in life. Several Danish twin studies have addressed this problem [VII; Stensballe et al., 2009; Poorisrisak et al., 2010]. First, a population study of 3–9-year-old twins (8,280 pairs) [VII] used information on hospital discharge diagnoses for severe RSV infection [Lynge et al., 2011] and on RSV infection diagnosed by antigen verification (enzyme linked immunosorbent assay of nasopharyngeal aspirate) [Stensballe et al., 2005], and information on asthma obtained from parent-reported questionnaires and from hospital discharge diagnoses [Lynge et al., 2011]. The study found good agreement between the two indicators for severe RSV infection (hospital-diagnosed and antigen-verified RSV infection, r=0.93) and also between the two indicators for asthma (hospital-diagnosed and parent-reported asthma, r=0.72) (Table 5). Using a common measure for severe RSV infection and for asthma, respectively, based on these different indicators for the two conditions, showed that the heritability of severe RSV infection and of asthma was 14% and 79%. The correlation between severe RSV infection and asthma was found to be higher in MZ than in DZ twin pairs (Table 5). In fact, it was shown that the association between severe RSV infection and asthma could be ascribed entirely to genetic effects shared between the two disorders with the genetic correlation not being significantly different from unity. Moreover, modelling the direction of causation between severe RSV infection and asthma showed that a model in which asthma was assumed to “cause” severe RSV infection fitted the data significantly better than did a model in which severe RSV infection was assumed to “cause” asthma. This conclusion was robust to adjustment for sex, birth weight and maternal smoking during pregnancy and is consistent with the hypothesis that severe RSV infection seems to be an indicator of the individual genetic susceptibility to asthma rather than a direct cause of asthma. Since the study was based mainly on registry information, it was not possible to resolve whether the subsequent pattern of asthma associated with a history of severe RSV infection was predominantly asthma associated with intermittent viral respiratory infections (transient wheeze) or whether there was an association with classical atopic asthma. However, the parent-reported prevalence of hay fever and atopic dermatitis was lower in asthmatic individuals with a history of severe RSV infection compared with those without a history of severe RSV infection. This indicates that IgE-mediated mechanisms play a less important role in the development of asthma in individuals with a history of severe RSV infection compared with those without such history. Second, the same population of twins was followed from birth until five years of age using time-to-onset data [Stensballe et al., 2009]. Asthma was diagnosed by hospital discharge diagnoses and by prescriptions of inhaled corticosteroids; and several additional confounding factors were adjusted for, such as type of delivery (vaginal vs. Caesarean section), gestational age, number of additional siblings, maternal age, maternal education, maternal income, parental cohabitation, and seasonality, using registry information [Lynge et al., 2011]. Severe RSV infection was found to be associated with a short-term increase in the risk of subsequent asthma, whereas asthma was associated with a long-term increased susceptibility to severe RSV infection, suggesting a host/genetic factor being responsible for the severe response to the RSV infection in individuals with asthma. Third, in a clinical follow-up study nested within the larger population of Danish twins, 3–9 years of age, 37 MZ twin pairs discordant for RSV hospitalisation at a mean age of 10.6 months were studied for the possible subsequent development of asthma, lung function impairment and atopy at a mean age of 7.6 years [Poorisrisak et al., 2010]. There were no differences observed between the RSV-hospitalised MZ twin and the non-hospitalised co-twin in any of the outcomes studied (asthma, wheezing, atopic dermatitis, airway responsiveness, positive SPT, FEV_1_, or FeNO), substantiating that severe RSV infection does not seem to directly cause asthma or other atopy-related conditions.

**Table 5:** Correlations between asthma and severe RSV infection in a sample of 8,280 Danish twin pairs, 3–9 years of age

	Parent-reported asthma	Hospital-diagnosed asthma	Antigen-verified RSV	Hospital-diagnosed RSV
All twins				
Parent-reported asthma	1			
Hospital-diagnosed asthma	0.72	1		
Antigen-verified RSV	0.34	0.42	1	
Hospital-diagnosed RSV	0.32	0.40	0.93	1
MZ twin pairs				
Parent-reported asthma	0.95			
Hospital-diagnosed asthma	0.57	0.81		
Antigen-verified RSV	0.32	0.41	0.77	
Hospital-diagnosed RSV	0.33	0.48	0.79	0.93
DZ twin pairs				
Parent-reported asthma	0.62			
Hospital-diagnosed asthma	0.31	0.51		
Antigen-verified RSV	0.18	0.21	0.76	
Hospital-diagnosed RSV	0.17	0.25	0.70	0.84

Correlations for all twins denote phenotypic (within-individual) correlations, whereas correlations for MZ and DZ twin pairs denote cross-twin correlations. All correlations are statistically significant (p<0.001). Modified from [VII].

It has been shown that up to 50% of hospitalisations for infant bronchiolitis are due to viruses other than RSV, particularly *rhinovirus* and *human metapneumovirus* [Singh et al., 2007]. Rhinovirus has been shown to be a major cause of infant bronchiolitis, probably as important as RSV, and coinfection with RSV has been linked to particularly severe illness [Papadopoulos et al., 2002; Singh et al., 2007]. Moreover, it has been shown that severe infection with rhinovirus or human metapneumovirus in infancy has a substantial impact on later asthma risk [Jackson et al., 2008]. These other types of viral infection have not been studied in twins in relation to asthma, and future twin studies should focus on a wider range of respiratory microbes, including viruses, in the search for early determinants of asthma.

## The fetal programming hypothesis

In 1989, Barker and colleagues observed that low birth weight was associated with death from ischaemic heart disease at adult age [Barker et al., 1989]. This observation gave rise to the *fetal programming (Barker) hypothesis*, the phenomenon whereby malnutrition and other adverse influences in utero permanently set the structure of different organs and the function of different key systems and through these mechanisms predetermine a person's risk of chronic diseases later in life [Barker et al., 2013]. There is a large body of experimental and epidemiological evidence that demonstrates this phenomenon, both in relation to cardiovascular and metabolic diseases, and also in relation to atopic diseases, autoimmune diseases, psychiatric diseases, and several cancers [Barker et al., 2013].

As for asthma, the relationship to birth weight is not straightforward. Turner summarized population studies of the relationship between birth weight and asthma published since 2000 and observed an inverse relationship between birth weight and asthma in 9 studies, 10 studies found no relationship, and 3 studies found a positive relationship, i.e. high birth weight was associated with asthma [Turner, 2012]. Inverse relationships were predominantly seen in large study populations (median=8,071 individuals), whereas studies reporting no effect tended to be of medium size (median=3,628 individuals), suggesting that study size may partly explain heterogeneity between earlier findings.

The basic premise of the fetal programming hypothesis in relation to asthma is that birth weight is used as an index of fetal wellbeing and the assumption is that adverse exposures regarding the respiratory or immune system are manifest as reduced growth. However, birth weight is the end point of nine months' growth, and insults at different gestational stages might result in different biological effects as tissues undergo critical developmental phases at different times; moreover, insults may result in *catch-up growth* associated with high birth weight, normal birth weight, or low birth weight [Turner, 2012]. Consequently, babies born with similar birth weights might have experienced very different antenatal environments and hence have very different risks of asthma, which may also explain disparities in earlier findings of the relationship between birth weight and asthma. Additionally, growth retardation may not always be associated with chronic disease later in life. Notably, rapidly growing fetuses are more vulnerable to inadequate nutrition because of the abrupt growth reduction this causes, whereas more slowly growing fetuses continue to grow during periods of undernutrition. Hence, downregulation of growth in early gestation may protect against undernutrition later in gestation [Poulsen, 2010].

Interestingly, twins are born an average of 1000g lighter than singletons and three weeks preterm [Kyvik, 2000]. Moreover, the intrauterine environment of twins differs from that of singletons in a number of ways that, according to the fetal programming hypothesis, could be speculated to put twins at a higher risk of asthma and other chronic diseases later in life. According to this hypothesis, MZ twins would be speculated to have an even higher risk of asthma compared with DZ twins because of a more hostile fetal environment, particularly for the smaller twin. MZ twins comprise one third of all twin pregnancies and result from cleavage of the ovum usually before day twelve after fertilisation. Two thirds of all MZ twin pregnancies are monochorionic and share a common placenta - they are either monoamniotic, which is rare, or diamniotic - whereas one third of all MZ twin pregnancies are dichorionic (and diamniotic), with two separate placentae (fused in about 15%). In contrast, DZ twins result from polyovulation and develop when two eggs released in the same menstrual cycle are simultaneously fertilised. DZ twins are always dichorionic (and diamniotic), with two placentae (fused in about 40%).

Differences in chorionicity and amniosity may explain differences in disease outcomes later in life. For example, in a recent Dutch twin cohort study, the median gestational age was one week longer in dichorionic than in monochorionic twins, and the mean birth weight was 221g higher [Hack et al., 2008]. Severe birth weight discordance (>20%) occurred more often in monochorionic than in dichorionic twins, and the incidence of necrotising enterocolitis was higher in monochorionic twins, after adjustment for age and birth weight. Further, there was a trend towards higher neuromorbidity in monochorionic twins. Similar findings were observed in an historical cohort of Dutch twins, which showed a higher mortality among monochorionic twins compared with dichorionic twins (27.7 vs. 15.8%) [Hack et al., 2006]. Interestingly, gestational age and birth weight were stronger predictors of perinatal mortality than chorionicity; and perinatal outcome was poorer for the second-born twin, especially in dichorionic twins. A third Dutch twin study found no difference in the risk of asthma between the first- and the second-born twin among vaginally delivered twins [van Beijsterveldt & Boomsma, 2008]. The same was observed among Australian twins, both for MZ as well as for DZ pregnancies [Duffy et al., 1990]. Effects of chorionicity and amniosity have not been studied in twins in relation to asthma, and future studies could address this to add insight into the role of the intrauterine environment in the aetiology of asthma. For example, in a twin study from Flanders, the heritability of blood pressure was not modified by chorionicity in MZ twins [Fagard et al., 2003]. Whether this would also apply in asthma is unclear.

Among Danish twins, the risk of parent- or self-reported asthma was slightly, albeit not statistically significantly, higher among MZ twins compared with DZ twins, particularly among children and young adults (aged 3–49 years), which would signal that MZ twins experience a more adverse fetal environment than do DZ twins [I]. However, postnatal factors may also be involved in explaining the difference in the risk of asthma between zygosity groups. An indication of this comes from the observation that, unlike in children and young adults, the risk of self-reported asthma among older adult Danish twins (50–71 years of age) was slightly *lower* in MZ twins compared with DZ twins, consistent with an increasing influence of other, possibly sociodemographic, risk factors throughout the lifespan that would differentiate MZ from DZ twins as a whole [I]. Moreover, seemingly the low birth weight and the adverse intrauterine environment of twins do not result in a higher risk of asthma in twins as a whole compared with singletons. Rather, there may be a physiological downregulation of fetal growth in twins starting in early gestation; and early embryonic development in twins may be timed slightly differently compared with singletons in order to avoid long-term negative effects of intrauterine growth retardation [Poulsen, 2010]. This has been exemplified in a few studies that have compared the prevalence of asthma in twins and singletons using similar methodology. Interestingly, a Swedish study of conscripts found a *reduced* risk of asthma in twins compared with singletons [Bråbäck & Hedberg, 1998], whereas a study from the UK found a reduced risk of hospitalisation for asthma among small twin children [Strachan et al., 2000]. An alternative interpretation of these studies is that twins are a special case of large family size, thereby supporting the theory that the opportunity for cross infection between twins may lead to an overall lower risk of asthma in twins as a whole. However, other factors, for example socioeconomic differences between twin and singleton families, may also explain these observations [Huovinen & Kaprio, 2001]; moreover, comparison of prevalence estimates in twins and singletons from other countries, ignoring diagnostic differences, seems not to reveal a different risk of asthma in twins in general compared with singletons [Thomsen et al., 2008c]. This is consistent with findings regarding other diseases and overall mortality in twins and singletons [Kyvik, 2000].

**Fig. 7:**
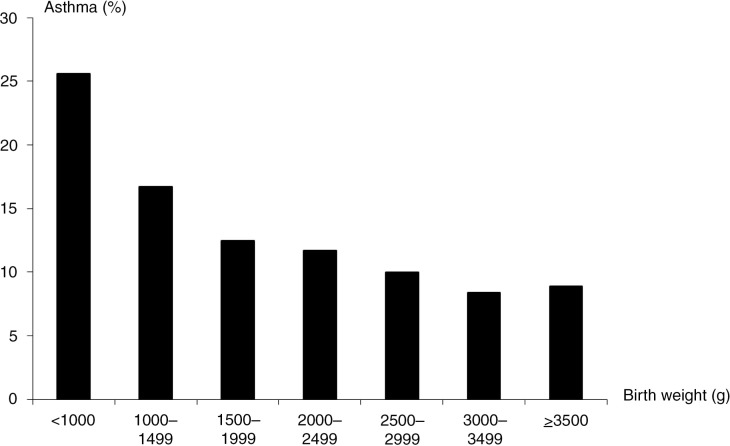
Relationship between asthma and birth weight in Danish twins, 3–9 years of age.

A population-based study of Danish twins (8,280 pairs) showed that children with a history of asthma at age 3–9 years weighed on average 122g less at birth compared with children who had not developed asthma [VIII]. This study used registry data on birth anthropometric factors obtained from midwives’ reports [Lynge et al., 2011] and therefore was not biased by lack of recall of birth weight by the mothers of the twins. There was a linear increase in asthma risk with decreasing birth weight; for every 100g *decrease* in birth weight the risk of asthma *increased* by 4% (Figure 7). Within twin pairs, the lower birth weight twin had a significantly *increased* risk of asthma compared with the heavier co-twin (11.3 vs. 9.9%) after adjustment for sex, birth length and Apgar score. Moreover, the matching of the twins in the co-twin control design allowed an inherent adjustment for other factors that would otherwise confound the association between birth weight and asthma such as gestational age, asthmatic predisposition, maternal age, maternal smoking, birth order effects, and upbringing. Therefore, the estimated risk of asthma can be assumed to reflect more precisely the influence of birth weight, or of the *asthmatic trajectory* represented by low birth weight. Notably, the risk of asthma tended to be *higher* in the lower birth weight MZ than DZ co-twin relative to the higher birth weight twin, especially for large intrapair differences in birth weight, suggesting that the relationship between low birth weight and asthma was mediated by *non-genetic* factors, and *not* by a common genotype underlying the association between low birth weight and asthma. This effect has also been observed among Finnish [Räsänen et al., 2000] and Swedish [Villamor et al., 2009; Ortqvist et al., 2009] twins, suggesting that low birth weight per se is not likely to be the causal factor leading to asthma. Instead, the association between low birth weight and asthma may be explained by early adaptation mechanisms in response to various adverse exposures in fetal life and early childhood. For example, infant weight gain, which is generally higher in children with low birth weight, has been associated with respiratory morbidity in childhood [Duijts, 2012]. A large population study of Dutch children showed that rather than fetal growth, weight gain acceleration in early infancy was associated with increased risk of asthma symptoms in preschool children [Sonnenschein-van der Voort et al., 2012]. Further, a large study of eight European populations found that children with a rapid body mass index (BMI) gain in the first two years of life had a higher risk of incident asthma up to age six years than did children with a less pronounced BMI gain in early childhood after adjustment for birth weight, weight-for-length at birth, gestational age, sex, maternal smoking in pregnancy, breastfeeding, and family history of asthma or allergies. In contrast, a rapid BMI gain at two to six years of age in addition to rapid gain in the first two years of life did not significantly enhance the risk of asthma [Rzehak et al., 2013]. These observations are in line with findings for diseases of *the metabolic syndrome* where rapid infant weight gain or rapid third trimester fetal weight gain has been associated with increased risk of obesity, type 2 diabetes and cardiovascular disease (*the thrifty phenotype hypothesis*) [Vaag et al., 2012], suggesting that similar pre-and perinatal mechanisms regulate the development both of asthma and other chronic diseases.

Other factors may also explain the increased risk of asthma in low birth weight children. One example is maternal smoking during pregnancy, which has been associated both with low birth weight and asthma in the offspring; children with reduced fetal growth are particularly vulnerable to maternal smoking during pregnancy [Bjerg et al., 2011] but, in contrast, the effect of maternal smoking during pregnancy on the risk of asthma seems to be only slightly reduced when taking into account fetal growth and preterm delivery, indicating that only a small fraction of the effect of maternal smoking on the risk of asthma is mediated through these pregnancy outcomes [Jaakkola & Gissler, 2004]. Maternal smoking may influence the risk of low birth weight and asthma through several mechanisms, for example via epigentic modification [van der Valk et al., 2012; Leslie, 2013], or via induction of T_H_2-polarisation [Singh et al., 2011]. However, these effects may apply both to low and normal weight infants. A second example is the heightened risk of several respiratory viral infections such as RSV in low birth weight infants that subsequently could unmask the child's risk for asthma [Rossi et al., 2007]. Interestingly, the risk of severe RSV infection is heightened in infants exposed to environmental tobacco smoke [von Linstow et al., 2008], which may also explain the subsequently higher risk of asthma. A third example is breastfeeding, which may have a differential effect on the risk of asthma according to birth weight. Specifically, breastfeeding seems to have a protective (or postponing) effect on asthma among children with high birth weight while it has no significant effect on asthma among children with normal or low birth weight [Xu et al., 2009]. Fourth, although unlikely, a common genetic origin of low birth weight and asthma cannot be completely ruled out. Several twin studies have attributed the relationship between low birth weight and later risk of type 2 diabetes to non-genetic causes [Poulsen et al., 1997; Grunnet et al., 2007]; nevertheless, recently, several genetic loci have been associated both with birth weight and type 2 diabetes and adult blood pressure in a large GWA study [Horikoshi et al., 2013].

Antenatal environmental factors could well influence the risk of asthma and atopic diseases in a complex developmental interplay with maternal and fetal immunogenetic factors. The development of the immune system begins early in fetal life. Lymphocytes derived from the yolk sac appear in the liver within several weeks of conception. By 10–12 weeks of gestation, they are evident in the thymus and show responsiveness to mitogen stimulation and allogeneic graft vs. host reactivity. Thymocytes appear to be capable of binding antigens from 20 to 22 weeks’ gestation, and allergen-specific responses have been recorded as early as 22 weeks’ gestation [Prescott, 2010]. Epigenetic mechanisms, i.e. the imprinting of (maternal) environmental experiences on infant gene expression, are thought to be at the root of developmental plasticity in relation to allergic disease [Kozyrskyj et al., 2011]. Specifically, there is an interest in factors that may promote allergic propensity by increased histone acetylation (T_H_2 promotion) and/or increased gene methylation (T_H_1 and regulatory T cell silencing). As an example, maternal folate supplementation (a dietary methyl donor) resulted in hypermethylation (suppression) of regulatory genes and the development of allergic disease in the offspring in an animal model [Hollingsworth et al., 2008]. The implication of this observation for human health is, however, not yet clear. Another example is the protective relationship between maternal n-3 polyunsaturated fatty acid consumption in pregnancy and subsequent infant allergic disease [Romieu et al., 2007]. This effect might also be mediated via epigenetic mechanisms or via microbial alterations to the maternal gut flora [Frei et al., 2012]. In fact there are several lines of evidence that now seem to link the fetal programming hypothesis to the hygiene hypothesis, providing a more coherent explanation of the rising trends in asthma and allergy. For example, maternal exposure to high microbial burden in German farming environments has been associated with altered expression of innate immune genes and reduced risk of allergic disease in the children [Ege et al., 2006]. Further, there is evidence to suggest that the apathogenic cowshed-derived microbial strain *Acinetobacter lwoffii* can mediate allergyprotective effects by epigenetic changes [Conrad et al., 2009]. Intranasal administration of this strain to pregnant mice was associated with significant effects on the ontogeny of splenic CD4+ T_H_1 interferon-γ production in the progeny. These differences were directly related to loss of histone 4 acetylation in the interferon-γ promoter [Brand et al., 2011], supporting that microbial exposure can modify fetal gene expression. It is likely that changes to the maternal gut microbiota during pregnancy have pronounced influences on fetal immune development. However, at present, the implication of these changes on the subsequent risk of allergic diseases in the infant is not clear and should therefore be subject to further study.

## The metabolic syndrome

The metabolic syndrome represents a cluster of cardiometabolic risk factors characterised by abdominal obesity, insulin resistance, dyslipidemia, and elevated blood pressure [Alberti et al., 2009]. The occurrence of the metabolic syndrome has risen to epidemic proportions during the past decades, and diseases associated with the metabolic syndrome, such as type 2 diabetes and cardiovascular disease, now pose serious health concerns in developed and, more recently, in developing countries [Eckel et al., 2005].

While the relationship between the metabolic syndrome and cardiovascular disease is well described, recent years have seen the identification of a link between the metabolic syndrome and other noncommunicable diseases, such as asthma, several cancers, gastroesophageal reflux disease, liver disease, neuropsychiatric diseases, psoriasis, and sleep disorders [Knight, 2011]. Indeed, asthma in the obese population may be considered a distinct clinical phenotype, characterised by later onset, female preponderance, greater symptomatology, decreased sensitivity to inhaled corticosteroids, and possibly also by a relatively low degree of eosinophilic inflammation compared with classical atopic asthma [Lugogo et al., 2010]. Interestingly, obese asthma patients may also experience more symptoms associated with exposure to indoor air pollutants such as fine particulate matter and nitrogen dioxide than normal-weight asthma patients [Lu et al., 2013].

Numerous population studies, both cross-sectional and longitudinal, in children and in adults, have shown a positive association between asthma and components of the metabolic syndrome, mostly obesity [Moreira et al., 2013, Papoutsakis et al., 2013]. There is a consistent but varying strength of association between obesity and asthma across populations, with a dose-dependent risk of asthma according to increasing BMI, even within the normal range of BMI, and with a stronger relationship to self-reported asthma and asthma symptoms compared with intermediate asthma phenotypes such as airway hyperresponsiveness and atopy. A systematic review and meta-analysis of *prospective* population studies found an increased risk of incident asthma in obese individuals, both among men (46% increased risk) and women (68% increased risk) [Beuther & Sutherland, 2007]. Further, a population study of Japanese adults suggested that Japanese persons may develop asthma at a lower BMI compared with individuals from Western populations [Fukutomi et al., 2012], which would indicate an influence of ethnicity-specific differences in the sensitivity to develop asthma in response to changes in BMI, consistent with Asian populations having a greater percentage of body fat at a given BMI compared with Western populations.

Type 2 diabetes has also been associated with asthma [Hashemzadeh & Movahed, 2009; Song et al., 2010] and asthma symptoms [Lee et al., 2009] in some population studies, but not in all [Rana et al., 2004], as has insulin resistance [Husemoen et al., 2008; Thuesen et al., 2009; Arshi et al., 2010]. In a recent large population study of ~85,000 adults from Spain, mutually adjusted analyses including all components of the metabolic syndrome and possible confounders showed that elevated waist circumference (or BMI), elevated serum triglyceride and low serum high density lipoprotein (HDL) were significantly associated with wheezing, and with stronger associations in individuals without concomitant rhinitis symptoms, i.e. in those who could be considered non-atopic [Fenger et al., 2013]. Finally, it has been shown that obese asthma patients show an improvement in symptoms of asthma, asthma severity, use of asthma medication, and pulmonary function after weight loss, surgical or non-surgical, indicating that the obese asthma phenotype is non-pharmacologically modifiable [Hakala et al., 2000; Stenius-Aarniala et al., 2000; Adeniyi & Young, 2012; Moreira et al., 2013].

Several hypotheses have been proposed to explain the association between obesity and asthma. First, obese individuals show changes in respiratory mechanics and physiology characteristic of asthma. Obese individuals tend to breathe at lower lung volumes [Delgado et al., 2008] and with a decreased cycling rate of airway smooth muscle, leading to decreased functional capacity, airflow limitation, mostly of a restrictive type [Nakajima et al., 2008], and increased airway responsiveness [Shore & Fredberg, 2005], although not all studies have been able to document this [Schachter et al., 2001; Sin et al., 2002]. Furthermore, obese asthma patients experience reduced pulmonary compliance due to fat compression and infiltration of the thorax and by an increase in lung blood volumes producing a subjective increase in dyspnoea [Delgado et al., 2008]. This mechanical/physiological hypothesis is attractive but fails to explain the documented difference in asthma risk according to subtle changes in BMI within the normal range of BMI.

A second hypothesis points to a detrimental effect of the chronic pro-inflammatory state associated with the metabolic syndrome and obesity, particularly abdominal obesity. There is an increased production of several inflammatory mediators - *adipokines* - in obese individuals, such as leptin, tumour necrosis factor alpha (TNF-α), IL-6, eotaxin, ghrelin, and C-reactive protein, while the production of the anti-inflammatory peptide adiponectin is reduced [Sood, 2010]. Specifically, leptin, discovered in 1994, is a well-studied adipokine that correlates positively to BMI and body fat percentage [Takeda et al., 2012]. Leptin receptors are expressed on T cells and on fetal and adult bronchial epithelial cells [Bergen et al., 2002]. Leptin influences satiety and basic metabolic rate, and studies have proposed a relationship between increased leptin production and a resulting inflammatory response dominated by eosinophils [Takeda et al., 2012]. Several studies [Guler et al., 2004; Sood et al., 2006], but not all [Kim et al., 2008; Jartti et al., 2009] have linked leptin to asthma. For example, a study of Turkish children showed that serum leptin levels were higher in asthmatic individuals compared with healthy individuals and significantly predicted having asthma independently of BMI, age, and sex [Guler et al., 2004]. A similar observation was made in a large cross-sectional study of adults from the United States in which serum leptin levels were significantly higher in persons with asthma, particularly in women, compared with those who had never had asthma, also after adjustment for triceps skinfold thickness and other covariates [Sood et al., 2006]. These studies suggest that leptin may provide a link between inflammation and T cell function in asthma. Furthermore, steroid hormones - estrogens - are produced in an excess amount in fat tissue by *aromatase* conversion of androgens [Tchernof & Després, 2013]. It has been shown that female sex hormones can modulate pulmonary inflammatory processes and influence airway responsiveness and lung function, thereby possibly playing a role in the development and exacerbation of asthma [Haggerty et al., 2003]. Notably, studies of women who receive postmenopausal hormone replacement therapy associate the effects of estrogens with the development of asthma [Troisi et al., 1995]. Moreover, early menarche, a condition with early increase in, and a consequently longer duration of exposure to, female sex hormones may be associated with development of asthma [Macsali et al., 2011] and has also been linked to both pre- and postmenarchal high BMI [Freedman et al., 2003]. Interestingly, the association between obesity and susceptibility [Castro-Rodríguez et al., 2001] to and severity [Varraso et al., 2005] of asthma may be more evident in women with early menarche.

A third hypothesis, which is supported by twin studies, suggests that shared genetic pathways for obesity and asthma account, at least in part, for the observed association between these conditions. Genetic variants associated with obesity have been shown also to be associated with asthma [Melén et al., 2010]. Interestingly, several polymorphisms (*3'UTR A/G* and *-2549A/G*) in the leptin gene have been associated with paediatric asthma [Szczepankiewicz et al., 2009]. Of further note are the β_2_-adrenergic receptor gene and the glucocorticoid receptor gene. The former encodes receptors involved in the regulation of airway tone and metabolic rate through sympathetic nervous system activity, whereas the latter encodes receptors that modulate inflammation in both asthma and obesity [Weiss, 2005]. Other variants of suggested pleiotropic importance are the TNF-α gene complex within which several polymorphisms have been associated with asthma, intermediate asthma phenotypes, and obesity [Li Kam Wa et al., 1999; Tantisira & Weiss, 2001]; and the low affinity immunoglobulin E receptor (FCɛRB) and uncoupling protein 2 and 3 genes that have been associated with measures of asthma and objective measures of atopy [Palmer, 2001], and fat distribution [Rosmond, 2003], respectively. Finally, several genes encoding various cytokines, such as *STAT6*, *IFNγ*, *IL1A*, and *LTA4H*, influence inflammatory processes in relation to both asthma and obesity [Weiss, 2005].

**Fig. 8:**
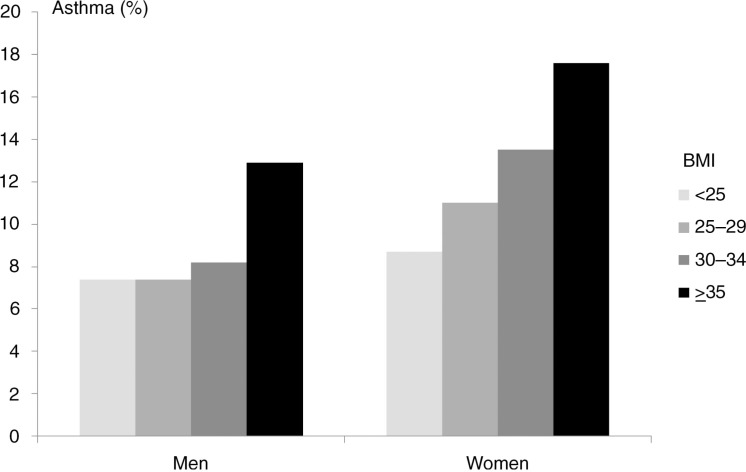
Relationship between asthma and BMI in Danish twins, 20–71 years of age.

The degree of the genetic association between asthma and components of the metabolic syndrome, primarily obesity, has been estimated in several twin studies. A Danish study of 34,782 twins, 20–71 years of age, addressing the relationship between asthma, BMI and type 2 diabetes is the largest and most comprehensive to date [IX]. In this study, questionnaire data on asthma were cross linked with data on self-reported BMI and with hospital discharge diagnosis data on type 2 diabetes [Lynge et al., 2011]. Unadjusted analyses showed that BMI was a significant predictor of asthma both in women and in men with a clear dose-response relationship between increasing levels of BMI and asthma (Figure 8). Furthermore, the risk of asthma was significantly higher in persons with type 2 diabetes compared with those without type 2 diabetes, both in women (16.6 vs. 9.6%) and in men (13.5 vs. 7.5%). After adjustment for age, smoking, chronic bronchitis, marital status, and zygosity, BMI remained a significant determinant of asthma in women but not in men, whereas type 2 diabetes was significantly associated with asthma both in women and in men, with no significant interaction between BMI and type 2 diabetes on the risk of asthma. In co-twin control analysis of the same population, asthma was more common in the twin with the highest BMI, and with an increased difference in the risk of asthma according to increasing BMI discordance within the twin pair, particularly in DZ twins, consistent with an underlying genetic relationship between obesity and asthma. The genetic correlation between BMI and asthma was estimated to be significant only among women (genetic correlation=0.15). A significant genetic correlation was also observed between type 2 diabetes and asthma (0.20), and, expectedly, also between BMI and type 2 diabetes (0.40).

Studying the subgroup of Danish twins aged 20–49 years, but on an occasion nine years earlier (when the twins were 12–41 years of age) showed a genetic correlation of 0.28 between obesity and asthma in women [Thomsen et al., 2007]. Despite differences in the usage of BMI as an obesity indicator (dichotomous vs. ordinal) between the two occasions, the results could indicate a reduction in the genetic association between obesity and asthma with age and/or a more marked genetic association between obesity and asthma in the more recent generations. A smaller twin study (1,484 pairs) of adults from the United States found a genetic correlation between BMI and asthma of 0.29 [Hallstrand et al., 2005], similar to the Danish studies. A small study of Chinese adults (483 pairs) found that sensitization to common allergens was positively genetically correlated with lipid levels (low levels of high density lipoprotein (HDL) and high levels of low density lipoprotein (LDL)), and percentage body fat, respectively, in men, but not in women [Ouyang et al., 2009]. However, genetic correlations were not statistically significant except for the association between sensitization and high LDL (genetic correlation=0.33).

It is a limitation of the twin studies of obesity and asthma that they used only self-reported data on asthma and BMI. Both men and women tend to overreport their height, and women, in particular, tend to underreport their weight with increasing level of overweight, which would lead to an underestimation of BMI among women [Boström & Diderichsen, 1997]. Further, even if correctly measured, BMI does not specify whether excess body mass is due to excess fat mass or how the body fat is distributed. This is problematic since especially central obesity is suspected to be associated with insulin resistance and asthma [Appleton et al., 2006]. Furthermore, twin studies have shown that the heritability of BMI differs from that of other obesity indicators, such as fat mass index and waist circumference, and also from serum leptin level [Hasselbalch et al., 2008]. Using these indicators of body fat would therefore possibly reveal different genetic correlations with asthma. As previous twin studies are limited by cross-sectional measures of BMI and asthma, future twin studies should employ a longitudinal framework to address their underlying genetic and environmental relationship.

The mechanical/physiological hypothesis, the inflammation hypothesis, and the genetic hypothesis explain the causes for the association between obesity and asthma, albeit possibly only in part. Indeed, the emerging interface between the hygiene hypothesis and the fetal programming hypothesis has led to the formulation of *the developmental origins of health and disease (DOHaD) hypothesis* as a universal paradigm that better encompasses the complex interactions between developmental factors, the (microbial) pre- and postnatal environment, and genetic factors leading to various chronic diseases, such as obesity and asthma, later in life [Blaser & Falkow, 2009; Ly et al., 2011]. Notably, both asthma and obesity often begin in early childhood, when the gut microbiota is primarily developed and when the infant immune system is shaped. There is evidence to suggest that a less diverse population of intestinal anaerobes in early life associates with both atopic diseases [Kalliomäki et al., 2001] and obesity [Turnbaugh et al., 2009], and evidence further suggests that the causes for alterations in the commensal microbiota, such as mode of delivery, breast-/formula-feeding habits, prematurity, dietary factors, and early antibiotic use, are common to obesity and asthma [Blaser & Falkow, 2009]. Similarly, fetal programming of obesity and asthma possibly also follows a common trajectory that seems to be linked to changes in gut microbiota [Vickers et al., 2007]. A study by Kalliomäki and colleagues found that children remaining normal weight at the age of seven years had a higher number of fecal Bifidobacteria and a lower number of Staphylococcus aureus in infancy compared with children who were overweight at age seven years [Kalliomäki et al., 2008]. Furthermore, the guts of obese individuals have reduced numbers of Bacteroidetes and increased numbers of *Firmicutes* compared with those of their lean counterparts [Ley et al., 2006]. Similarly, depletion of Bifidobacteria and Bacteroidetes and abundance of Staphylococcus aureus have been linked to a higher risk of asthma [Russell et al., 2012]. A randomized trial of pre- and postnatal maternal administration of *Lactobacillus rhamnosus* beginning four weeks before expected delivery and continuing for six months after delivery indicated that probiotics might modify the growth pattern of the child by restraining the excessive weight gain that occurs in the first two years of life but not that between age two to four years [Luoto et al., 2010]. This is an interesting finding, since excessive weight gain, particularly in the first two years of life, has also been associated with increased risk of asthma [Rzehak et al., 2013].

Taken together, multiple genetic, metabolic and immunological factors possibly act in a developmental context with the pre- and postnatal environment to link the metabolic syndrome to asthma. Experimental and epidemiologic data suggest that gut microbial diversity early in life, rather than specific microbial strains, is likely the key factor in promoting normal immune development. Well designed birth cohort studies with extensive data on neonatal and maternal vaginal/gut microbiota, (epi)genetic modifications, and immune responses are needed to further delineate the underlying immune modulation by gut microbiota important in the development and prevention of asthma and obesity [Ly et al., 2011]. It would be interesting to explore these relationships in prospective twin cohorts as twins provide a means of controlling various confounding or modifying variables, such as genetic factors as well as pre-and postnatal environmental exposures, for example maternal and neonatal diet, antibiotic use, and other undisclosed, but potentially important, factors relating to neonatal life and upbringing.

## Conclusions

The Danish Twin Registry is one of the largest and most well structured in the world. The studies originating from this registry have contributed substantially to our understanding of the aetiology of asthma. In particular, based on extensive nationwide questionnaire surveys, national hospital discharge registries, and clinical and paraclinical data, the studies outlined in this thesis have shown that asthma is a highly heritable disease with genetic factors accounting for around 70% of the variation in its susceptibility. However, the fraction of susceptibility accounted for by genetic factors diminishes over the life span, particularly among men. Moreover, unlike most previous twin studies, common environmental factors (factors that increase the similarity between household members) were found also to contribute significantly to the susceptibility to asthma. Notably, small size of previously studied twin populations and methodological factors alluding to the twin design itself may have led to inflated estimates of asthma heritability, and this must be kept in mind when interpreting the results of these studies.

While most previous twin studies have focused on the variation in the *susceptibility* to asthma, a novel contribution of the Danish twin studies is the estimation of the heritability of several aspects of the clinical expression of the disease such as the variation in *age at onset* and *symptomatology*. These qualities of asthma were found also to be influenced by genetic factors, to some extent. However, in contrast to the high heritability of asthma susceptibility, genetic factors account for only around 35% of the variation in the age at onset and for around 25% of the variation in the overall symptomatic severity of the disease and for even less of the variation in the severity of individual asthma symptoms such as wheezing, shortness of breath, chest tightness and cough. Moreover, the genetic factors that influence these individual symptoms, respectively, were found to have little overlap, highlighting a substantial genetic heterogeneity with the clinical expression of asthma.

The heritability as well as the genetic and environmental correlations between *intermediate asthma phenotypes* were other novel focuses of the Danish twin studies. It was shown that variation in key intermediate asthma phenotypes, such as airway responsiveness (sensitivity to inhaled methacholine), airway inflammation (level of exhaled nitric oxide), lung function (FEV_1_ and FVC, and the ratio between these), as well as atopy and serum total IgE, was also influenced to a large extent by genetic factors; however, with evidence of considerable genetic heterogeneity between these traits. Specifically, it was shown that these traits each had a heritability of at least 50% (some even higher, for example the heritability of serum total IgE was 81%), but that methodological factors such as sample size and ascertainment of twins for clinical studies may have led to inflated estimates of heritability also of these traits. An exception to the high heritability of intermediate asthma phenotypes was HDM sensitivity, of which genetic factors explained only 6% of the variation in susceptibility, indicating that overall atopy is far more heritable than atopy to specific allergens. Furthermore, among Danish twins, it was shown that specific asthma symptoms correlated only to a small extent with these intermediate traits in respect to genetic and environmental factors, suggesting that aetiological factors associated with asthma symptoms and objective asthma traits, respectively, differ. Finally, asthma was shown to be strongly associated with the other atopic diseases, i.e. atopic dermatitis and hay fever, and this association was to a large extent ascribable to genetic factors common to the three atopic diseases.

Serial cross-sectional studies of Danish twins allowed an investigation of the possible change in relative importance of genetic and environmental effects on asthma over time. Specifically, it was shown that among Danish adolescent twins, the prevalence of asthma *increased* from 7.1 to 10.8% between 1994 and 2003, whereas, in the same period, the heritability of asthma also increased significantly from 79 to 91%. This was particularly due to an increased concordance for asthma among MZ twins in 2003 compared with 1994, whereas the concordance for asthma among DZ twins was more or less unchanged. This supports the hygiene hypothesis, i.e. the prevalence of asthma has increased over the past decades in reaction to widespread environmental changes - changes thought to be related to a decreased microbial diversity in Western societies. Predominantly, the influence of genetic factors seemed to have increased over time as a result of environmental changes (gene-environment interaction).

Another observation that fits well with the hygiene hypothesis is that among Danish twins, atopic dermatitis, and to a lesser extent asthma, and type 1 diabetes were shown to be inversely related; atopic dermatitis was about five times less common in individuals with type 1 diabetes compared with individuals without type 1 diabetes. These diseases, which could be regarded as T_H_2 and T_H_1 immune-mediated diseases, respectively, were shown to share environmental factors to a sizable extent (environmental correlation=0.52), but in contrast, be negatively genetically correlated (genetic correlation=−0.30).

Danish twin studies of infant broncholitis caused by RSV showed that respiratory viral infections encountered early in life constitute an exception to the general rule that microbial stimulation confers a protective effect on asthma development. Particularly, severe infant RSV infection was shown to be associated with an increased risk of asthma due to a shared genetic predisposition of RSV infection and asthma, substantiating the theory that severe RSV infection seems to be an indicator of the individual genetic susceptibility to asthma rather than a direct cause of asthma.

It was shown that Danish twin children with a history of asthma at age 3–9 years weighed, on average, less at birth compared with children who had not developed asthma, with a linear increase in asthma risk with decreasing birth weight. Within twin pairs, the lower birth weight twin had a significantly increased risk of asthma compared with the heavier co-twin. Of note, the risk of asthma was higher in the lower birth weight MZ than DZ co-twin relative to the higher birth weight twin, especially for large intrapair differences in birth weight, suggesting that the relationship between low birth weight and asthma was mediated by non-genetic (environmental) factors, and not by a common genotype underlying the association between low birth weight and asthma.

Finally, it was shown that among Danish adult twins, asthma was significantly related to components of the metabolic syndrome such as obesity and type 2 diabetes. Specifically, the risk of asthma increased linearly in relation to increases in BMI with an almost two times increased risk of asthma in individuals, particularly women, with a BMI above 30 kg/m^2^. Further, the risk of asthma was about doubled in subjects with type 2 diabetes. The genetic correlation between BMI and asthma was estimated to be significant only among women (genetic correlation=0.15). A significant genetic correlation was also observed between type 2 diabetes and asthma (genetic correlation=0.20).

In conclusion, the Danish twin studies have substantiated several leading hypotheses, such as the hygiene hypothesis and the fetal origins hypothesis, as explanations for the modern disease epidemic that apart from asthma also involves autoimmune diseases and the metabolic syndrome. These studies have pointed to a common origin of these chronic inflammatory diseases tracing back to fetal life and early childhood and have revealed that the aetiology of asthma must be understood and further explored in the context of the dynamic cross-talk between genetic factors, (antenatal) developmental factors, and the modern (changing) environment.

### Future perspectives

Many questions regarding the aetiology of asthma remain unanswered. Particularly, in order to identify novel biological factors for pharmacological targeting and to develop robust diagnostic and prognostic clinical applications, ongoing efforts to investigating the genetic basis of asthma are mandatory. However, recent years’ gene-hunting efforts have translated very little into successful preventive strategies and new treatments for asthma and have revealed that most of the genetic variation of asthma is due to variants with relative risks of less than 1.2, and with each variant explaining only a fraction of a percent of liability [Gibson, 2009]. In contrast, the epidemic aspects of asthma and of other common inflammatory diseases implicate recent widespread environmental changes. Moreover, the importance of antenatal and early life (non-genetic) risk factors lends further support to our understanding of asthma as a multifactorial developmental phenotype.

As modern genomic medicine advances, twin studies now endow new implications [Visscher, et al., 2008]. A key area of future research where twin studies are expected to be particularly valuable is epigenetics [Bell & Spector, 2011; Bell & Saffery, 2012]. Epigenetic mechanisms, which play an essential role in regulating transcription, are possibly capable of explaining various non-Mendelian features such as the relatively high degree of discordance of asthma in MZ twins. Studying tissue samples, for example blood or airway cells, from MZ twin pairs discordant for asthma provides a unique opportunity for exploring the influence of epigenetic factors on disease expression. However, this area of research is still in its infancy and only few studies of twins have been performed. For example, among MZ twin pairs discordant for asthma, exposure to second-hand smoking is associated with modifications in both regulatory T cells and effector T cells at the transcriptional level among asthmatic individuals, suggesting a differential function of T cell subsets in MZ twins discordant for asthma regulated by changes in DNA methylation [Runyon et al., 2012]. Similarly, in vitro allergen challenge of MZ twin pairs discordant for allergic rhinitis revealed significant differences in mRNA and protein levels between the allergic and healthy twins [Sjogren et al., 2012]. It would be very interesting to further study the epigenetic aspects of asthma, preferably by using larger samples of well-characterised twins.

Another area where twin studies, particularly studies of discordant twins, can be expected to be valuable is in the uncovering of the role of the human microbiome in relation to asthma. Twin studies have revealed that host factors, possibly genetics, are involved in determining the diversity of the gut microbiome [Reyes et al., 2010] and also that considerable individual non-genetic variation exists even between closely related individuals [Turnbaugh et al., 2009]. In relation to disease prediction, a study of Malawian twins showed that the gut microbiome might be a causal factor in kwashiorkor [Smith et al., 2013]; previously frozen fecal communities from twin pairs discordant for kwashiorkor were transplanted into *gnotobiotic* mice. The combination of Malawian diet and kwashiorkor microbiome produced marked weight loss in recipient mice, accompanied by perturbations in amino acid, carbohydrate, and intermediary metabolism that were only transiently ameliorated with a ready-to-use therapeutic food [Smith et al., 2013]. Another study of twin pairs discordant for Crohn's disease revealed a significantly higher faecal bacterial diversity in the healthy twin compared with the co-twin with Crohn's disease [Dicksved et al., 2008]. Likewise, it has been shown that individual twin survivors of adolescent/young adult Hodgkin lymphoma have a deficit of rare gut microbes compared with their co-twin controls [Cozen et al., 2013]. Further work in twins is needed to determine whether these effects also pertain to asthma and, particularly, whether reduced microbial diversity is a consequence of disease, its treatment, or a particularly hygienic environment.

An area of research where twin studies probably could also prove valuable is in the understanding of the role of the barrier surfaces, particularly the skin, airways, and gut in relation to asthma development and progression. The advent of *FLG* mutations as a link between atopic dermatitis and asthma indicates a causal relationship between these diseases in the context of a deficient skin barrier and challenges the finding among twins that the relationship between atopic dermatitis and asthma is mainly genetic in origin. It would be interesting to address this *leaky barrier hypothesis* further in the context of defects in various other structural epidermal and epithelial (airway or gut) proteins. Specifically, other epithelial barrier proteins may have implications for asthma development, also in the absence of atopic dermatitis. For example, membrane expression of *caveolin-1* has been shown to be significantly lower in airway epithelium from asthma patients than from non-asthmatic individuals [Hackett et al., 2013]. Importantly, reduced caveolin-1 expression is accompanied by loss of junctional E-cadherin and *β-catenin* expression, disrupted epithelial barrier function, and increased levels of the pro-allergic cytokine *thymic stromal lymphopoietin* [Hackett et al., 2013]. Twin pairs discordant for barrier protein gene mutations would be interesting to study in relation to asthma, whereas, for example, MZ twin pairs concordant for gene mutations associated with epithelial barrier dysfunction would serve as an interesting sample for studying gene-environment interaction in atopic diseases. Moreover, twins would enable an estimation of the magnitude of variance of asthma susceptibility ascribable to mutations in such barrier protein genes.

Finally, twin studies have shown that the antenatal environment plays a key role in determining asthma susceptibility. Notably, low birth weight, which can be considered a marker of fetal malnutrition and an adverse intrauterine environment, has been linked to asthma development. Additionally, several environmental exposures, such as maternal tobacco smoke and diet, have been shown to modify the child's risk of asthma through various effector mechanisms. However, our current knowledge of these mechanisms is very limited and awaits further study. Samples of amniotic fluid from monoamniotic and, in particular, diamniotic twin pregnancies constitute a possibly valuable resource for determining the role of the antenatal environment in the aetiology of asthma. For example, among singletons it has been shown that there are detectable differences between asthmatic and non-asthmatic children already in utero (prior to elective Caesarean section) in the concentration of various proteases found in amniotic fluid [Turner et al., 2013]. The amniotic fluid is a complex and dynamic milieu that changes as pregnancy progresses [Underwood et al., 2005]. It contains cell derivates from many fetal systems such as the airways, skin, gastrointestinal tract, and urinary system. It provides mechanical cushioning and antimicrobial effectors that protect the fetus, and its constituents comprise multiple nutrients and growth factors such as carbohydrates, proteins (more than a hundred unique proteins have been identified in the amniotic fluid proteome [Harman, 2008]), lipids, electrolytes, enzymes, and hormones that facilitate fetal growth [Underwood et al., 2005]. It would be interesting to examine twin pregnancies with information on environmental (maternal) exposures and twin-to-twin differences in amniotic constituents in relation to atopic disease outcomes. Further, as other complex disorders such as obesity and type 2 diabetes have been linked to antenatal factors in similar ways as asthma, it would also be interesting to elucidate the role of the fetal environment in the obesity-asthma epidemic. Indeed, the most challenging task in future asthma research will be to try to integrate the various identified risk factors - (epi)genetic, developmental, and environmental - in the explanation of the origins of asthma and of related common complex diseases and, further, to try to process this information preventively and therapeutically. Twin studies provide a valuable means in this demanding task.

## Danish summary

Formålet med studiet var i en landsdækkende population af tvillinger i alderen 3–71 år at undersøge forskellige årsagsmæssige aspekter ved astma. Idet der blev anvendt såvel spørgeskemadata, data fra Landspatientregistret, samt kliniske data, blev der specifikt fokuseret på at bestemme betydningen af genetiske og miljøbetingede faktorer for henholdsvis tilbøjeligheden til astma, debutalderen for astma samt for variationen i den kliniske sværhedsgrad af astma herunder for variationen i *intermediære* astmafænotyper. Yderligere undersøgtes sammenhængen mellem astma og en række perinatale risikofaktorer, primært fødselsvægt, samt sammenhængen mellem astma og forskellige andre inflammatoriske sygdomme, herunder atopisk eksem og høfeber, type 1 diabetes, respiratorisk syncytial virus (RSV) infektion samt fedme og type 2 diabetes.

Konkordansen for astma blandt monozygote tvillinger var omkring dobbelt så høj som blandt dizygote tvillinger. Forskellen i konkordans var dog alders- og kønsafhængig med en generelt højere forskel blandt yngre personer, især mænd, mens forskellen hos ældre var betydelig mindre. Heritabiliteten af astma blev fundet til at være omkring 70%, dog væsentlig lavere blandt ældre voksne, hvor forskelle i risiko overvejende var forklaret ved fælles miljøfaktorer. Blandt børn og yngre voksne fandtes, at heritabiliteten i to uafhængige populationer med samme aldersfordeling øgedes med omkring 15% over en observationsperiode på ni år, hvilket kunne være foreneligt med en ændret ekspression af gener grundet ændringer i generelle miljøpåvirkninger i løbet af observationsperioden. Variationen i debutalderen for astma var under indflydelse af genetiske faktorer, idet der sås en højere korrelation mellem monozygote tvillinger end mellem dizygote tvillinger for dette tidspunkt, især blandt mænd. Denne forskel i korrelation var mere udtalt i familier hvor astma debuterede i en tidlig alder, som udtryk for at astma med debut i barnealderen har en større genetisk komponent end astma med debut senere i livet. Heritabiliteten af debutalderen for astma blev bestemt til at være 34%. Sværhedsgraden af astma bedømt ved frekvensen af luftvejssymptomer var ligeledes under en vis genetisk indflydelse med en heritabilitet på 24%. Endvidere fandtes variationen i sværhedsgraden af specifikke astmasymptomer i form af hvæsende vejrtrækning og åndenød at være under indflydelse af genetiske faktorer.

Variationen i objektivt målte intermediære astmafænotyper var under væsentlig genetisk indflydelse. Især for niveauet af total IgE i serum fandtes en heritabilitet på 81%, mens heritabiliteten af nitrogenoxid i udåndingsluften (FeNO) var 67%. For bronchial reaktivitet målt ved følsomheden for inhaleret methacholin samt graden af luftvejsobstruktion målt ved forholdet mellem forceret ekspiratorisk volumen i det første sekund (FEV_1_) og forceret vital kapacitet (FVC) fandtes heritabiliteter på henholdsvis 43% og 22%. Heritabiliteten af sensibilisering overfor husstøvmide var derimod kun 6%, hvilket var foreneligt med en betydelig større miljømæssig komponent for sårbarheden overfor at udvikle specifikke allergier end for den generelle tendens til atopi (som havde en heritabilitet på 54%). Der fandtes et vist genetisk overlap mellem de enkelte intermediære fænotyper, men overvejende var variationen i hver af disse under indflydelse af specifikke genetiske faktorer. Den kliniske sværhedsgrad af astma målt som sværhedsgraden af luftvejssymptomer var kun i ringe grad korreleret til de objektivt målte intermediære astmafænotyper, og der fandtes en tilsvarende lav og insignifikant korrelation mellem genetiske faktorer for kliniske symptomer og disse objektive træk, navnlig atopi.

Lav fødselsvægt blev fundet at være en risikofaktor for udvikling af astma i barnealderen. Således påvistes det, at blandt tvillingpar med forskellig fødselsvægt var det oftest tvillingen med den laveste fødselsvægt, som senere udviklede astma. Forskellen var mest udtalt blandt monozygote tvillingpar sammenlignet med dizygote tvillingpar tydende på, at sammenhængen mellem fødselsvægt og astma er betinget af miljøfaktorer.

Astma var stærkt associeret med høfeber og atopisk eksem, og dette var overvejende forklaret ved fælles genetiske effekter; korrelationen mellem astma og høfeber var for 70% vedkommende forklaret ved fælles genetiske effekter, mens korrelation mellem astma og atopisk eksem for 81% vedkommende var forklaret ved fælles genetiske effekter.

Astma fandtes at have markante genetiske fællestræk med alvorlig luftvejsinfektion med RSV i de tidlige barneår. Især syntes den genetiske tilbøjelighed til astma at være en forløber for udvikling af alvorlig luftvejsinfektion, mens det modsatte fandtes at være mindre sandsynligt, nemlig at alvorlig luftvejsinfektion var årsag til udvikling af astma.

Astma fandtes ikke at være signifikant relateret til type 1 diabetes hverken blandt børn eller voksne, selvom tidligere studier har antydet en invers sammenhæng mellem disse sygdomme, primært blandt børn. I modsætning hertil påvistes en signifikant invers genetisk sammenhæng mellem atopisk eksem og type 1 diabetes tydende på modsatrettede genetiske mekanismer for atopiske (T_H_2-medierede) og autoimmune (T_H_1-medierede) sygdomme. Samtidig fandtes miljøfaktorer for atopisk eksem og type 1 diabetes at være positivt korrelerede.

Forekomsten af astma var øget hos voksne personer med henholdsvis type 2 diabetes og forøget BMI, navnlig kvinder, og denne sammenhæng kunne til en vis grad tilskrives genetiske fællestræk.

Sammenfattende viste studiet, at astma har en betydelig arvelig komponent, primært hvad angår den generelle tilbøjelighed til sygdommen, men også i et vist omfang hvad angår mere specifikke fænotypiske træk såsom debutalderen og sværhedsgraden af sygdommen samt en række objektive træk i form af luftvejsreaktivitet, luftvejsobstruktion, luftvejsinflammation samt allergisk tilbøjelighed. Astma har tydelige genetiske fællestræk med andre atopiske sygdomme, såsom atopisk eksem og høfeber, samt med alvorlige virusinfektioner i luftvejene i den tidlige barndom. Samtidig synes astma hos voksne at have ætiologiske fællestræk med komponenter af det metaboliske syndrom eksemplificeret ved fedme og type 2 diabetes. Studiet peger desuden på en række fælles miljøbetingede årsagsfaktorer for inflammatoriske sygdomme, herunder astma, relateret til fostertilværelse og tidlig opvækst med baggrund i moderne vestlig levevis.

Gennem studier af tvillinger er det vist, at det er muligt at opnå nuanceret viden om årsagsforhold ved astma samt om sammenhængen med beslægtede sygdomme. Fremtidige genetisk epidemiologiske studier af astma bør inddrage denne viden i bestræbelserne på at afdække de mere specifikke årsager til sygdommens opståen og forløb.
